# Community matters: stress tolerance and survival of *Bacillus subtilis* and *Staphylococcus capitis* in a synthetic space habitat consortium

**DOI:** 10.3389/fmicb.2026.1869903

**Published:** 2026-07-27

**Authors:** Carolin Luisa Krämer, Yen Ly-Sauerbrey, Milina Maier, Luis Kadler, Anna Rehm, Bernd Walkenfort, Claudio Neidhöfer, Stefan Leuko, Oliver Schwengers, Mike Hasenberg, Stella Marie Timofeev, Stefan Janssen, Kristina Beblo-Vranesevic, Katharina Döscher-Siems

**Affiliations:** 1 Department of Natural Sciences, University of Applied Sciences Bonn-Rhein-Sieg, Rheinbach, Germany; 2 Department of Applied Biology, Aerospace Microbiology, Institute for Aerospace Medicine, Cologne, Germany; 3 Algorithmic Bioinformatics, Justus-Liebig University, Giessen, Germany; 4 Imaging Center Essen, Electron Microscopy Unit, Faculty of Medicine, University of Duisburg-Essen, Essen, Germany; 5 Division of Clinical Bacteriology and Mycology, University Hospital of Basel, Basel, Switzerland

**Keywords:** co-cultivation, microbial community, resistance, space microbiology, stress tolerance, synthetic consortia

## Abstract

**Introduction:**

Conventional microbiological methods typically examine isolated bacterial species, which limits the insight into how microorganisms behave in more realistic, complex ecosystems. In contrast, synthetic bacterial consortia offer a practical and ecologically meaningful model for studying species interactions and responses to stress. These interspecies dynamics, both cooperative and competitive, can significantly influence the survival and physiology of individual species, revealing patterns that single-species tests often miss. Exploring microbial resilience under stress is vital for advancing space microbiology and ensuring the functionality of enclosed environments like the International Space Station (ISS) and in the future in spacecraft traveling to the Moon or Mars.

**Methods:**

To assess the stress tolerance of bacterial species within a consortium, a synthetic bacterial consortium was constructed using species representative of the spacecraft microbiome, including genera such as *Bacillus, Pseudomonas*, and *Staphylococcus*. The stress tolerance of *Bacillus subtilis* and *Staphylococcus capitis* was evaluated both individually and within the defined consortium under spaceflight-relevant conditions, including desiccation, X-ray irradiation, and hydrogen peroxide exposure. Survival rates were quantified by colony-forming unit (CFU) counts. Co-cultivation approaches were employed to assess biofilm formation and growth within the consortium. Consortium dynamics following stress exposure and cultivation were further investigated using 16S rRNA gene sequencing.

**Results:**

The stress tolerance of *B. subtilis* and *S. capitis* differed between individual exposure and consortium conditions. Hereby, the consortium context influenced stress responses in a stressor-specific manner. Co-cultivation experiments demonstrated observable growth in various combinations of consortium members, with differences in early-stage biofilm formation and CFU counts noted. Investigations of consortium dynamics revealed a statistically significant difference before and after incubation indicating that over time the consortium reaches a stabilized relative abundance profile.

**Discussion:**

Our findings underscore the importance of considering community-level interactions when evaluating bacterial stress tolerance. Synthetic bacterial consortia represent a valuable approach to bridging the gap between reductionist and systems-level microbiology, offering critical insights for both terrestrial biotechnology and the advancement of space exploration.

## 1 Introduction

Microorganisms impact every ecosystem they inhabit, ranging from environments on our planet to extraterrestrial habitats such as the International Space Station (ISS) in low Earth orbit (LEO). Regardless of the extent of cleaning, some microorganisms will inevitably stay in the respective environment. Even cleanrooms, which are meant to reduce contamination, have their own distinct microbiome (Mora et al., 2016a). This is also true for the ISS, whose microbiome is dominated by human-associated microorganisms and “adaptation specialists” (Mora et al., 2016b). While the ISS microbiome is shaped by its environment (Rai et al., 2021), the microbiome also affects the wellbeing of their inhabitants (Kelly et al., 2022; Hoisington et al., 2015). The advent of next-generation sequencing has enabled a more comprehensive understanding of the extraordinary richness and diversity of microbial life. Microorganisms typically exist within microbial consortia, which are groups of diverse microorganisms, including bacteria, archaea, fungi, and viruses, that coexist and interact within specific niches or environments (Fuhrman, 2009). These consortia exhibit various interactions, such as mutualism, competition, and commensalism (Großkopf and Soyer, 2014). Microbial consortia regulate a wide range of complex processes, including wastewater treatment for chemical contaminant reduction, and the microbial communities within the human gut are essential for digestion and gut health (Wu et al., 2019; Rowland et al., 2018).

However, most experimental investigations to date have focused on individual microbial species, and elucidating the complexity of microbial communities continues to present significant challenges. Even relatively simple natural communities often consist of more than 10 distinct members (Curtis et al., 2002). Due to the complexity of naturally found consortia, experimental designs use synthetic microbial consortia as surrogates, which can be artificially engineered to analyze key features, such as interactions and survival of natural communities. Synthetic microbial consortia can be designed in a bottom-up approach, meaning the consortium is built by combining species with known characteristics, or in a top-down approach where the consortium is a simplified version of a respective natural community (Großkopf and Soyer, 2014). Synthetic microbial consortia can aid in studying microbial interactions in a simplified, predictable environment, or can reduce variability compared to more complex natural microbial communities. With the help of synthetic microbial consortia, microbial interactions such as competition, cooperation, or signaling can be investigated, which can affect stability, metabolic efficiency, and resistance (Sun et al., 2023; Wang et al., 2025a,b). Resistance and survival of microorganisms are strongly influenced by the ecological context in which they exist (Bottery et al., 2020). One very special ecological niche is the indoor environment of space stations. Conditions in space stations imposed on microorganisms include microgravity, higher ionizing radiation levels, exposure to desiccation and cleaning agents. Some studies have shown that under spaceflight conditions virulence and resistance toward antibiotics was increased in *Enterobacter bugandensis* (Sengupta et al., 2024; Singh et al., 2018), biofilm formation was enhanced under spaceflight conditions in *Pseudomonas aeruginosa* (Kim et al., 2013), and microorganisms from the ISS might have adapted to their environment in space and showed enhanced biofilm formation (Mora et al., 2016b; Szydlowski et al., 2024). This is particularly relevant due to the unique conditions in space, which have a negative effect on the astronauts' health by reducing their immune functions (Demontis et al., 2017; Afshinnekoo et al., 2020). However, other studies have found that while prevalence of antibiotic resistance genes and virulence factors were not increased on the ISS, present microorganisms showed adaptation to the unique environmental conditions (Mora et al., 2016b, 2019).

Sporulation constitutes a critical microbial survival strategy in spacecraft-associated environments. Endospore-forming Firmicutes, particularly *Bacillus* species, are frequently identified in spacecraft assembly cleanrooms and on the ISS due to their remarkable resistance to desiccation, radiation, oxidative stress, and nutrient limitation (Tirumalai et al., 2013; Kempf et al., 2005; Vaishampayan et al., 2012). *Bacillus* spores endure extended exposure to extreme environmental conditions through mechanisms such as reduced metabolic activity, specialized spore structures, low water content, and robust DNA protection and repair systems (Chiang et al., 2019; Tirumalai et al., 2018, 2013; Nicholson et al., 2012). Owing to their persistence and significance for forward contamination, spore-forming microorganisms are central to planetary protection and space microbiology research (Danko et al., 2021; Kempf et al., 2005).

Bacterial resistance to environmental stressors can be a key factor in assessing the risk of microbial contamination, which in turn aids in mitigating risks. When studied as single species in isolation, microorganisms may exhibit different survival characteristics than when part of a microbial consortium. These characteristics may also depend on the microbial consortium itself, which could influence their adaptability, resilience to environmental fluctuations, and access to diverse resources (Wang et al., 2023). Consequently, survival of single species toward stressors may vary significantly compared with the survival of species within a consortium. Bacteria embedded within microbial consortia experience a dynamic network of interactions that can either enhance or diminish survival (Adekoya et al., 2025; Srinivasan et al., 2024; Stubbendieck et al., 2016). For instance, co-exposure within a bacterial consortium can increase survival of species toward environmental stressors, including pesticides, antibiotics, and fungicides (Smith et al., 2024). Cooperative mechanisms such as resource sharing, cross-protection against stress, and cell-to-cell signaling often confer collective advantages, enabling consortia to withstand hostile conditions more effectively than individual species (Scheuerl et al., 2020). At the same time, competitive interactions for nutrients and space can suppress certain members, shaping community composition and stability. These complex interplays highlight that bacterial survival is not just a function of individual capacity but emerges from the balance of cooperation and competition within the broader microbial consortia (Wilson et al., 2017; Xu, 2021). Additionally, horizontal gene transfer can enable microorganisms to better survive unfavorable conditions (Zhu et al., 2023). This is also highlighted by a recent study which found that single bacteria were less resilient to antimicrobial copper-coated surfaces compared to the same species exposed to the surfaces within a bacterial consortium (Ly-Sauerbrey et al., 2025). Another study investigating a microbial community under simulated microgravity and low-dose ionizing radiation, found that the diversity and number of Bacilli in the synthetic community increased under simulated space environment (Lu et al., 2023). Hence, to assess risk of microbial contamination, studying resistance of species as single species and imposed in a microbial consortium is a promising approach. Bacterial survival under stress conditions involves distinct physiological strategies, such as endospore formation in taxa like *Bacillus* and non-spore dormancy states, including viable-but-non-culturable or non-replicative persistent states. These dormancy states are particularly significant for culture-based detection methods, as viable cells may not form colonies under standard recovery conditions. In actinobacteria, including *Micrococcus luteus*, dormancy and resuscitation processes can involve mechanisms associated with resuscitation-promoting factors (Rpf) (Mali et al., 2017; Tirumalai et al., 2025; Havis et al., 2019).

In this study, we developed a synthetic bacterial consortium which is based on prominent members of the ISS microbiome, and tested two of the species for both their survival exposed as a single species and within the bacterial consortium. In a comparative approach we investigated resistance of *Staphylococcus capitis* and *Bacillus subtilis*, which have been commonly detected on the ISS, toward desiccation, X-ray irradiation and hydrogen peroxide. Further, experiments on pairwise co-cultivation, biofilm formation of the single species, and the bacterial consortium were conducted. Additionally, pilot studies on the consortium dynamics before and after cultivation as well as after stress exposure were performed. Studying single-species vs. consortium survival is crucial, as it reveals both the fundamental limits of bacterial resilience and the emergent properties of microbial consortia, including their functions in ecosystems and engineered environments.

## 2 Materials and methods

### 2.1 Used species and growth conditions

For cultivation of the species of the bacterial consortium different media were prepared including tryptic soy yeast broth (TSYB: 17 g/L casein peptone, 3 g/L soy peptone, 5 g/L NaCl, 3 g/L yeast extract, 2.5 g/L K_2_HPO_4_, 2.5 g/L glucose, pH 7.3), tryptic soy yeast agar (TSYA: 17 g/L casein peptone, 3 g/L soy peptone, 5 g/L NaCl, 3 g/L yeast extract, 2.5 g/L K_2_HPO_4_, 2.5 g/L glucose, 15 g/L bacteriological agar, pH 7.3), brain heart infusion (BHI, 5 g/L beef heart infusion, 12.5 g/L calf brain infusion, 2.5 g/L disodium hydrogen phosphate, 2 g/L glucose, 10 g/L peptone, 5 g/L sodium chloride) broth and agar (supplemented with 15 g/L bacteriological agar), double concentrated Luria-Bertani Miller (2 × LB, 20 g/L peptone, 20 g/L NaCl, 10 g/L yeast extract) broth and agar (supplemented with 15 g/L bacteriological agar). The bacterial species were incubated in the respective cultivation medium at the respective incubation temperature and time given in Table 1.

**Table 1:** Bacterial strains of synthetic bacterial consortium.

Bacterial species	Strain	Isolation site	DSM number	References	INSDC accession	Cultivation medium	Incubation temperature	Incubation time
*Staphylococcus capitis*	K1-2-2-23	ISS, V2A Steel	DSM 111179	Sobisch et al., 2019	GCA_025272695	TSYB/TSYA	37 °C	16–24 h
*Bacillus subtilis*	W2-B1-K4	ISS, Touch Array Brass (CuZn37)	–	Krämer et al., 2025	GCA_057654875.1	TSYB/TSYA	37 °C	12–16 h
*Pseudomonas antarctica*	CMS 35^T^	Antarctica	DSM 15318^T^	Reddy et al., 2004	GCA_010634845	TSYB/TSYA	Ambient	12–16 h
*Streptococcus halotolerans*	HTS9 ^T^	Respiratory Tract of *Marmota himalayana*	DSM 101996^T^	Niu et al., 2016	GCA_001598035	2xLB, TSYB/TSYA	37 °C	24 h
*Propionibacterium cyclohexanicum*	TA-12 ^T^	Spoilt Orange Juice	DSM 16859^T^	Kusano et al., 1997	GCA_900111365	BHI, TSYB/TSYA	37 °C	24 h
*Micrococcus luteus*	W4-Al-K1	ISS, Touch Array Aluminum Case	-	Krämer et al., 2025	GCA_057816565.1	NB, TSYB/TSYA	37 °C	24 h
*Acinetobacter radioresistens*	50V1	Preflight Mars Odyssey Orbiter	–	Mccoy et al., 2012	GCA_002834555.1	TSYB/TSYA	37 °C	16–18 h

The strains which were used for the bacterial consortium are given with their reference source and DSM number if available, isolation site, and cultivation media, incubation temperature and time. Additionally, the International Nucleotide Sequence Database Collaboration (INSDC) accession number is given of published genomes. All strains were either purchased from the German Collection of Microorganisms and Cell Cultures (Leibniz Institute DSMZ) or retrieved from archived isolates generated or used in previous work at the German Aerospace Center (DLR), Cologne. ^T^ is standing for type strain.

#### 2.1.1 Preparation of bacterial suspension and consortium

For the preparation of bacterial suspensions, 20 mL of the respective medium according to the species was inoculated with the respective species and incubated at the respective incubation temperature and time (Table 1). Grown cultures were then transferred to 50 mL tubes and centrifuged at 4,000 × g for 10 min. The supernatant was discarded and the cell pellets were washed twice by adding phosphate buffered saline (PBS: 7 g/L Na_2_HPO_4_, 3 g/L KH_2_PO_4_, 4 g/L NaCl, pH 7.5) and subsequently centrifuging at 4,000 × g for 10 min. After washing, the cells were resuspended in PBS, and the optical density at 600 nm (OD_600nm_) was measured to adjust the cell number in the single bacterial suspensions. Bacterial suspensions of single species were prepared with an OD_600nm_ of 0.125 if not stated otherwise. To create the synthetic bacterial consortium, equal amounts of adjusted bacterial suspensions were added together in one tube for a final OD_600nm_ of 0.125.

### 2.2 Scanning electron microscopy

For scanning electron microscopy (SEM) glass discs with a 12 mm diameter were sterilized with UV-C irradiation for 60 min before inoculation. One glass disc each was added per well to a 24-well plate and 600 μL of the respective bacterial suspension was added. Afterwards, the plate was incubated statically at 37 °C for 24 h. Afterwards, IMCES sampling protocol was applied: Cells were fixed by replacing the medium with 4% formaldehyde, 2.5% glutaraldehyde in 0.1 M PHEM buffer (60 mM PIPES, 25 mM HEPES, 10 mM EGTA, 2 mM MgCl_2_) and incubated at 4 °C for 24 h. For long-term storage, 1 × fixative was replaced with a 1% formaldehyde solution in 0.1 M PHEM buffer.

For imaging of the samples, IMCES routine protocols were applied. Imaging was performed using a Zeiss Crossbeam 540 operating at a tension of 3 kV with a beam current of 1 nA. Images with a pixel size of 5 nm were acquired using the Zeiss acquisition software Smart SEM (Carl Zeiss Microscopy GmbH, Germany).

### 2.3 Whole genome data

Whole genome data of *P. antarctica, S. halotolerans, P. cyclohexanicum, S. capitis*, and *A. radioresistens* are publicly available on NCBI. DNA extraction and subsequent whole genome sequencing of *B. subtilis* was performed by Microsynth AG (Microsynth AG, Switzerland). Whole genome data of *M. luteus* has been already submitted to NCBI as part of another study, which is currently undergoing peer review, the accession number will be updated during the review process. Whole genome data of *B. subtilis* has been submitted to NCBI. The INSDC accessions are given in Table 1.

For annotation of genomes, Bakta v1.11.0 (Schwengers et al., 2021) through Bakta Web (Beyvers et al., 2025) was used. Potential biosynthetic gene clusters for secondary metabolites were retrieved using Galaxy (25.1.1.dev0) via antiSMASH (Blin et al., 2025). Additionally, biosynthetic gene clusters were identified by analyzing each genome with GECCO v0.9.10 (Genome-wide Enzyme-Centric COmposition) using default settings (Carroll et al., 2021).

### 2.4 Co-cultivation approaches

For determination of growth in co-cultivation, 500 μL TSYB were added per well of a 48-well plate and the respective single species or combinations of the consortium was added in a 1:1,000 dilution of a previously prepared bacterial suspension with an OD_600nm_ of 0.05 to reach a final OD_600nm_ of 0.00005. Growth of cultivation approaches was measured using oCelloScope™ (BioSense Solutions ApS, Denmark). Before co-cultivation and after co-cultivation for 24 h, CFU/mL of *B. subtilis* and *S. capitis*, respectively, were determined as described in Section 2.6. All co-cultivation approaches were performed as three independent experiments with three biological replicates each. Before and after incubation for co-cultivation the pH was determined using pH-Fix 4.5–10.0 color-fixed pH test strips (Macherey-Nagel, Germany).

### 2.5 Crystal violet biofilm formation assay

Biofilm formation was investigated using the semi-quantitative crystal violet assay according to Stepanović et al. (2000) with the following modifications. Crystal violet biofilm formation in the following refers to the quantification of biomass. For investigation of biofilm formation of single species, 500 μL of prepared bacterial suspensions adjusted to an OD_600nm_ of 0.125 resuspended in TSYB were added to a 24-well plate and incubated statically for 24 h at 37 °C. For investigation of biofilm formation of co-cultivation approaches, bacterial suspensions of each species were adjusted to an OD_600nm_ of 0.05 and added in pairwise combinations for a final OD_600nm_ of 0.1 with subsequent static incubation for 24 h at 37 °C. After incubation, the medium was carefully discarded, and the potential biofilm was washed twice with 300 μL PBS. After removal of PBS, the plate was left to dry for 10 min. To dye the biofilm, 200 μL of 0.5% crystal violet (Merck, Germany) in 25% methanol was added and incubated for 30 min in the dark. Afterwards, crystal violet was removed, and cells were washed twice with ddH_2_O before adding 300 μL 95% ethanol to dissolve the crystal violet stain. The plate was then placed on a shaker for 5 min. Subsequently, the absorbance at 570 nm was measured in a microplate reader (Infinite M200 PRO, Tecan, Switzerland). The crystal violet assay was performed in three different experiments with three biological replicates.

### 2.6 Co-exposure to stressors

For co-exposure assays, cells suspensions were prepared as described in Section 2.1.1. To determine survival after co-exposure, CFU per mL (CFU/mL) were determined by plating a 10-fold dilution. For determination of survival of *S. capitis*, cell suspension after treatment was plated onto TSYA plates supplemented with 5 μg/mL rifampicin. For determination of survival of *B. subtilis*, treated cell suspensions were plated on TSYA plates supplemented with 50 μg/mL streptomycin. Previously tested antibiotic resistance of species of bacterial consortium is given in Supplementary Table 1. To determine survival fractions, the cell count after exposure (N) was divided by the initial cell count (N_0_). All co-exposure stress assays were performed in three independent experiments with three biological replicates each. It has to be noted that, CFU measures correspond to culturable surviving cells under the tested recovery conditions, and hence may underestimate viable cells that entered non-culturable or dormant states.

#### 2.6.1 Desiccation assay

To analyze the desiccation tolerance of *S. capitis* and *B. subtilis* as single species and within a synthetic consortium, bacterial suspensions were prepared as previously described (Section 2.1.1). Five sterile 96-well plates were prepared as follows: For both species- *S. capitis* and *B. subtilis*- and the bacterial consortium, six wells each were filled with 20 μL of the prepared cell suspensions. Additionally, six wells were filled with 20 μL PBS each as a negative control. For the initial cell count, 180 μL TSYB were added directly to each well and cell count was determined. For desiccation, plates were left to dry under a sterile laminar flow bench overnight. After 1, 7, 14, 21, and 28 days, respectively, dried cells were resuspended in 200 μL TSYB and survival fractions were determined as described previously. Additionally, after resuspension, well plates were incubated at 37 °C and OD_600nm_ was measured every 20 min in a microplate reader (Infinite M200 PRO, Tecan, Switzerland) for 18 h after orbital shaking for 5 s.

#### 2.6.2 X-ray irradiation

For X-ray irradiation 100 μL of prepared cell suspensions (Section 2.1.1) were transferred into sterile PCR tubes. X-ray irradiation was performed as described in Cortesão et al. (2020). Samples were irradiated with X-rays (200 kV, 15 mA) with cumulative doses of 50, 100, 250 and 500 Gy using a closed X-ray system (RS225, Gulmay, Germany) with an average dose rate of 15.3 Gy/min ± 1.7 Gy/min. Determination of dose rate was performed by placing a cut-open PCR tube on top of a TM30013 ionization chamber, which was connected to a UNIDOS^webline^ dosimeter (PTW, Germany). For determination of survival, the initial cell count of an untreated cell suspension (0 Gy) was determined.

#### 2.6.3 Exposure to hydrogen peroxide

To determine tolerance toward hydrogen peroxide treatment, cell suspensions were prepared as described previously (Section 2.1.1). For exposure to hydrogen peroxide with a final concentration of 1.5% and 3%, 100 μL of 15% and 30% hydrogen peroxide solution were added, respectively, to 900 μL of cell suspension. To stop exposure to hydrogen peroxide after 45 min and 90 min, respectively, 30 μL were taken out of each well, and added promptly to 270 μL of a 1 mg/mL catalase solution in PBS.

### 2.7 Determination of consortium shifts using 16S rRNA sequencing

To determine shifts in the bacterial consortium, DNA extraction was conducted from the bacterial consortium after stress exposure. Per stress condition, three 200 μL samples each of the bacterial consortium were frozen immediately after exposure to the different stressors. Additionally, triplicates of 200 μL of the bacterial consortium after stress exposure were incubated at 37 °C for 18 h and subsequently stored at −20 °C until DNA extraction. An incubation time of 18 h was chosen for cultivation, as this time period was a trade-off between growth of the different species so that it allowed species to progress beyond lag and exponential growth without several species remaining in plateau phase for too long.

#### 2.7.1 DNA extraction

For DNA extraction the ZymoBIOMICS 96 MagBead DNA Kit (ZymoResearch, Germany) was used. For cell lysis, 200 μL of the respective sample, 550 μL lysis buffer, and 2 μL ZymoBIOMICS Spike-in Control I (ZymoResearch, Germany) were added into Bashing Bead Tubes. Bashing Bead Tubes were added to a Tissue Lyser (Qiagen, Germany) and mechanically lysed at 30 Hz for 5 min. Subsequently, samples were centrifuged at 10,000 × g for 1 min and 220 μL per sample were added to a deep-well 96-well plate. Further DNA extraction was performed with the help of the epMotion 5075 pipetting robot (Eppendorf, Germany).

#### 2.7.2 16S rRNA gene sequencing

For 16S rRNA gene sequencing, the Quick-16S NGS Library Prep Kit (ZymoResearch, Germany) was used according to the manufacturer's protocol using Quick-16S Primer Set V3-V4 for targeting the 16S V3-V4 (341f-806r) region. ZymoBIOMICS Microbial Community DNA Standard was used as positive control and molecular grade water as negative control. For targeted sequence amplification, 40 cycles were used. After barcoding, 100 ng of product for each sample were used for normalization and were included in the final library pool. Concentration was measured using Qubit (Thermofisher Scientific, Germany) and adjusted to 4 nM. Library denaturing and MiSeq sample loading was performed using Illumina MiSeq Reagent Kit v3 (600 cycles) (Illumina, USA) according to the manufacturer's manual loading 570 μL denatured, diluted library (12 pmol) and 30 μL denatured, diluted PhiX control v3 (12 pmol) (Illumina, USA).

#### 2.7.3 Bioinformatic analysis of consortium dynamics

Basecalling was performed with bcl2fastq (v2.19.0.316), and cutadapt (Martin, 2011) (v1.18) was used to trim adapter sequences and generate fastq files. With fastqc and multiqc (Ewels et al., 2016), quality control was conducted. Processing was performed using Qiita (Gonzalez et al., 2018) (ID 16283): library split for demultiplexing, trimming to 150 bp and ASV creation with deblur (Amir et al., 2017) (v2021.09). Using QIIME2 (Bolyen et al., 2019) (v2025.4) we assigned taxonomy to the ASVs with the Greengenes2 2024.09 release (Mcdonald et al., 2024), using the Greengenes (v13.8) release (Mcdonald et al., 2012) to filter out mitochondria and chloroplasts. Additionally, reads with an occurrence of < 10 over all samples were filtered out as well. The feature table was rarefied to a rarefaction depth of 50,000 features per sample. For beta-diversity, weighted and unweighted UniFrac (Lozupone et al., 2011) and Bray-Curtis Dissimilarity (Bray and Curtis, 1957) were calculated, and visualized through a PCoA as an EMPeror plot (Vázquez-Baeza et al., 2013).

Significant differential abundance analysis was carried out using the ANCOM (Mandal et al., 2015) plugin from QIIME2.

We compiled a set of full length 16S rRNA sequences for all seven consortium members from the obtained genome data (see Section 2.3). As some isolates contain multiple copies of the 16S rRNA gene, we clustered all sequences via “cd-hit” (version 4.8.1, options -c 0.99 -T 2 -d 999 -p 1 -g 1 -aS 0.95 -A 50 -uL 0.05 -uS 0.05 -sc 1) (Fu et al., 2012). Considering reverse and reverse complement versions of full length 16S rRNA sequences, we selected one representative sequence per cluster by (a) cluster size (larger is better) (b) being flagged by cd-hit as representative and (c) preferring original sequence orientation. Representative sequences (with their isolate annotation) were used to compile a small blast database via “makeblastdb” (version 2.16.0+, -dbtype nucl) to query all ASV sequences against this database via “blastn” (version 2.16.0+, -task blastn -outfmt “6 std qlen slen nident qcovs” -max_target_seqs 5). The isolate taxon name was assigned to an ASV if blast returned a result with e-value < 10^10^, 5 mismatches at most, pident at least 97% and at most 10 bp of ASVs missing in blast hit. ASVs not covered by this process were taxonomically assigned to GG2v2022.10 via the Qiime2 Naive Bayes classifier (version qiime2-amplicon-2024.10) (Bolyen et al., 2019).

### 2.8 Statistical analysis

Statistical analysis for determination of statistical significance was performed using Sigmaplot 14.5 (Inpixon GmbH, Germany).

To determine statistical significance of co-cultivation experiments including biofilm formation and CFU count, Shapiro–Wilk tested normality and Brown-Forsythe tested equal variances. If Shapiro–Wilk failed, a Kruskal–Wallis One Way Analysis of Variance (ANOVA) on ranks was performed. If Brown-Forsythe failed, a Kruskal–Wallis One Way ANOVA was also performed. When Kruskal–Wallis ANOVA showed significance, Dunn's method was used as a multiple comparison procedure against a control group.

To compare CFU counts after exposure as single species and after co-exposure within the consortium, a Shapiro–Wilk test checked normality and a Brown-Forsythe test checked equal variances. If both tests passed and equal variances were assumed, a Student's *t*-test determined significance of survival after highest exposure with detected survival. If Shapiro–Wilk failed, a Mann–Whitney Rank Sum Test was performed.

## 3 Results

### 3.1 Mapping of bacterial consortium

To design a bacterial space habitat consortium, isolates obtained from the ISS or spacecraft-associated facilities, which are among commonly isolated and detected genera on the ISS as well as representative biosafety level 1 strains from commonly observed genera were selected (Mora et al., 2019; Checinska et al., 2015; Checinska Sielaff et al., 2019). An overview of the used strains is given in Table 1.

#### 3.1.1 Morphological analysis

For a visual overview of the species' morphology and early surface attachment, SEM images of the single species (Figure 1) and after cultivation within the whole consortium were prepared (Figure 2). SEM images revealed morphological distinctions among the examined species. *S. capitis* and *M. luteus* displayed characteristic cocci-shaped cells, with *S. capitis* frequently appearing in tightly associated clusters. *B. subtilis, P. cyclohexanicum*, and *P. antarctica* exhibited expected rod-shaped morphology, with *B. subtilis* showing surface-associated aggregates. *A. radioresistens* showed coccobacilli-shaped cells, distinguishable from more spherical cocci and elongated rods (Figure 1). However, due to insufficient growth of *S. halotolerans* after static incubation at 37 °C for 24 h, no images of *S. halotolerans* could be included. Differently shaped cells were visible within the bacterial consortium and have been marked to indicate the putative species (Figure 2). Figures 2A, C display various surface areas where the consortium was incubated, highlighting differences in distribution. Close-up images in 3-fold magnification are given in Figures 2B, D, respectively, and are indicated by a white box. Close-up images illustrate putative matrix formation and interaction or at least spatial closeness of the different species. Figure 2E represents an agar plate with the whole consortium incubated.

**Figure 1:**
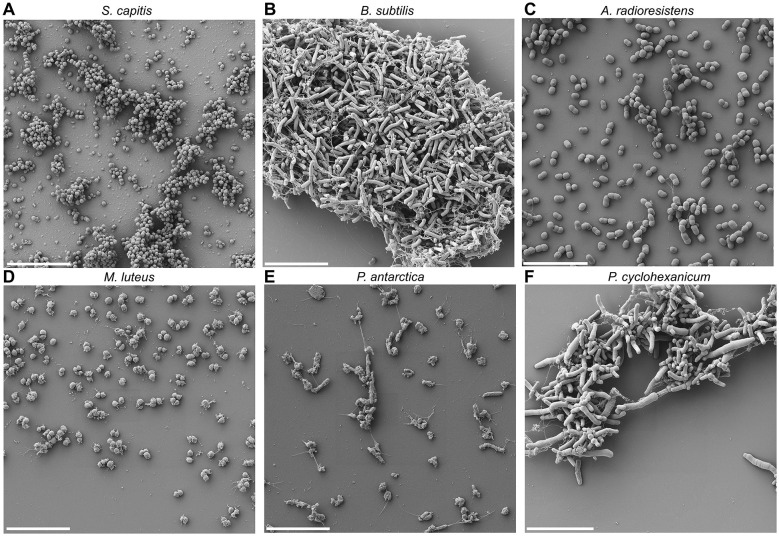
SEM images of selected space habitat consortium species after static incubation at 37 °C for 24 h. **(A)** *S. capitis*, **(B)** *B. subtilis*, **(C)** *A. radioresistens*, **(D)** *M. luteus*, **(E)** *P. antarctica*, **(F)** *P. cyclohexanicum*. Scale bar corresponds to 10 μm.

**Figure 2:**
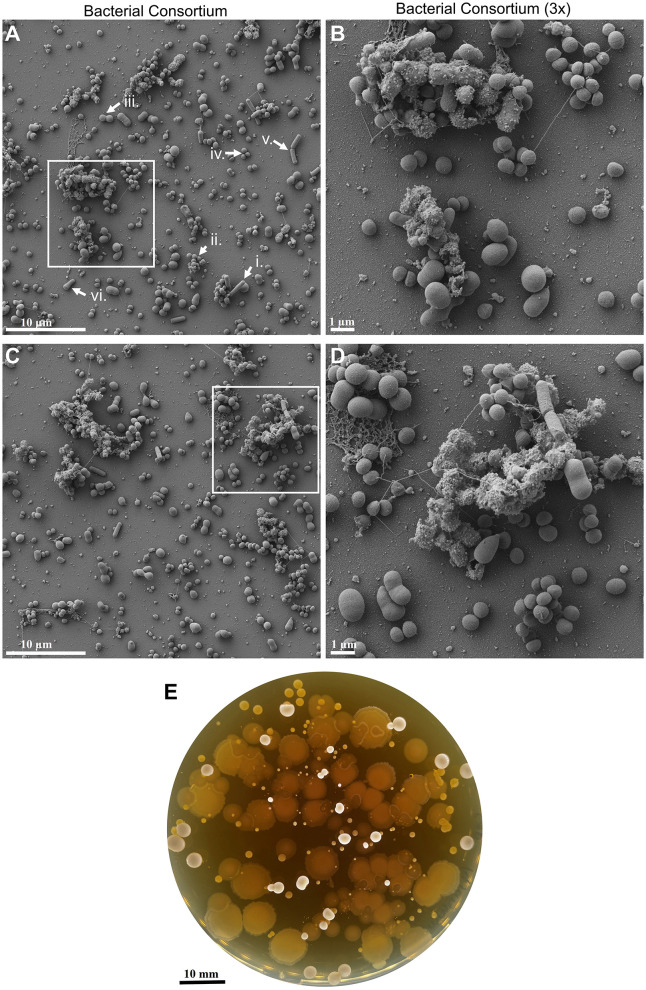
SEM images of space habitat consortium after static incubation at 37 °C for 24 h. **(B, D)** Bacterial consortium (3 × ) on the right represents 3-fold magnified images of the consortium, compared to **(A, C)** the bacterial consortium images on the left. Morphotypes consistent with species are indicated by (i) *B. subtilis*, (ii) *S. capitis*, (iii) *A. radioresistens*, (iv) *M. luteus*, (v) *P. antarctica*, (vi) *P. cyclohexanicum*. **(E)** TSYA plate with the bacterial consortium after incubation.

#### 3.1.2 Genotypic analysis

Additionally, whole genome sequences of the consortium members were obtained to identify potential gene clusters for the production of secondary metabolites (Supplementary Tables 2, 3). The genome of *B. subtilis* exhibited the greatest number of potential gene clusters for secondary metabolite production, encompassing various peptide antibiotics such as bacilysin, bacillibactin, bacillaene, and fengycin, in addition to RiPPs (ribosomally synthesized and post-translationally modified peptides). These RiPPs display diverse biological activities including antimicrobial properties. In addition, other gene clusters for the production of RiPP have been discovered. The genome of *A. radioresistens* harbored potential gene clusters for siderophores and aryl polyene. The genome of *M. luteus* contained gene clusters responsible for the production of ectoine, β -lactone, and siderophores. The genome of *P. antarctica* harbored gene clusters for the production of β -lactone, siderophores, NRPS (non-ribosomal peptide synthetase), and aryl polyene. The genome of *P. cyclohexanicum* contained gene clusters responsible for the production of NRPS and terpenes. The genome of *S. halotolerans* was found to contain gene clusters that are capable of producing RiPP-like products, and a polyketide synthase.

Additionally, we analyzed the genomes to determine their potential for quorum sensing. Within the genome of *B. subtilis* we found a gene encoding for a lactonase putatively involved in quorum sensing. In the genome of *S. capitis*, we identified *agrA, agrC*, and *agrD*, and a gene cluster putatively responsible for the production of a cyclic-lactone autoinducer.

### 3.2 Co-cultivation approaches

Co-cultivation of pairwise combinations could aid in disentangling increasingly complex interaction layers within bacterial consortia. Utilizing single species growth as a baseline enables the identification of specific partner-dependent effects, such as growth inhibition or enhancement, which may be obscured in more complex consortia. Hence, as first co-cultivation approaches, we investigated growth of the single species, pairwise combinations of *B. subtilis* and *S. capitis*, respectively, and growth of the whole consortium (Figures 3, 4). The CFU of *B. subtilis* and *S. capitis* before co-cultivation was determined, but we did not find statistically significant differences between cultivation as single species, respectively, and in co-cultivation (Supplementary Figure 1). Additionally, determination of the pH-value of the different co-cultivation combinations at the beginning and end of the incubation time revealed no statistically significant changes in pH (Supplementary Figure 2).

**Figure 3:**
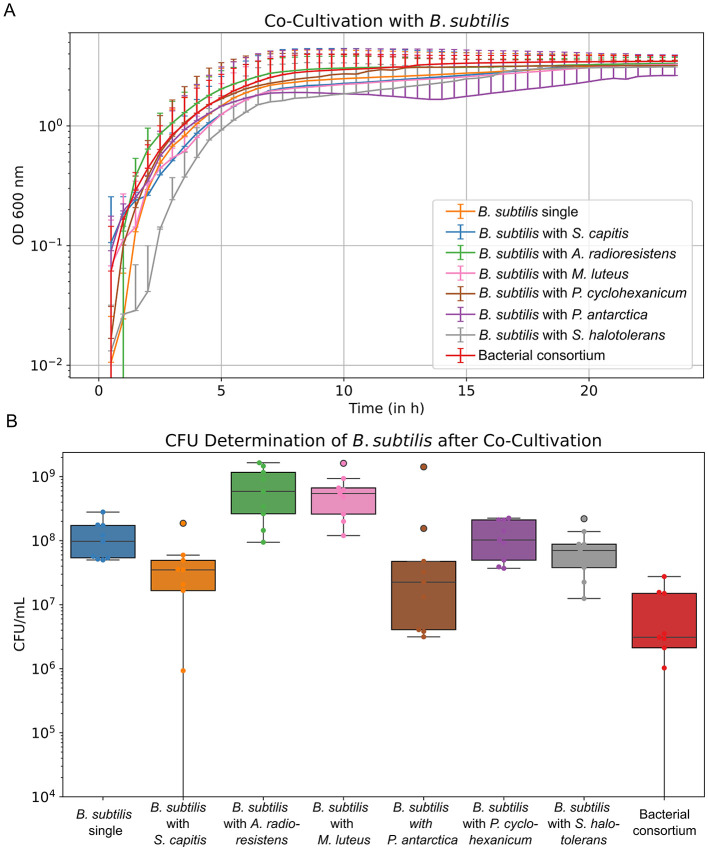
Co-cultivation approaches with *B. subtilis*. **(A)** Growth curve of *B. subtilis* as single culture and in combination with each member of the bacterial consortium, and of the bacterial consortium as a whole. Combinations with *B. subtilis* are given as “with species name.” **(B)** Determination of CFU/mL of *B. subtilis* after 24 h of co-cultivation in combination with each different species of the bacterial consortium and within the bacterial consortium. Significance between CFU/mL of *B. subtilis* when incubated as single species and in co-cultivation is indicated by asterisks (*p* < 0.05^*^). Error bars crossing the x-axis indicate no detectable culturable recovery for replicates of co-cultivation with *S. capitis* (*n* = 1), and the bacterial consortium (*n* = 1).

**Figure 4:**
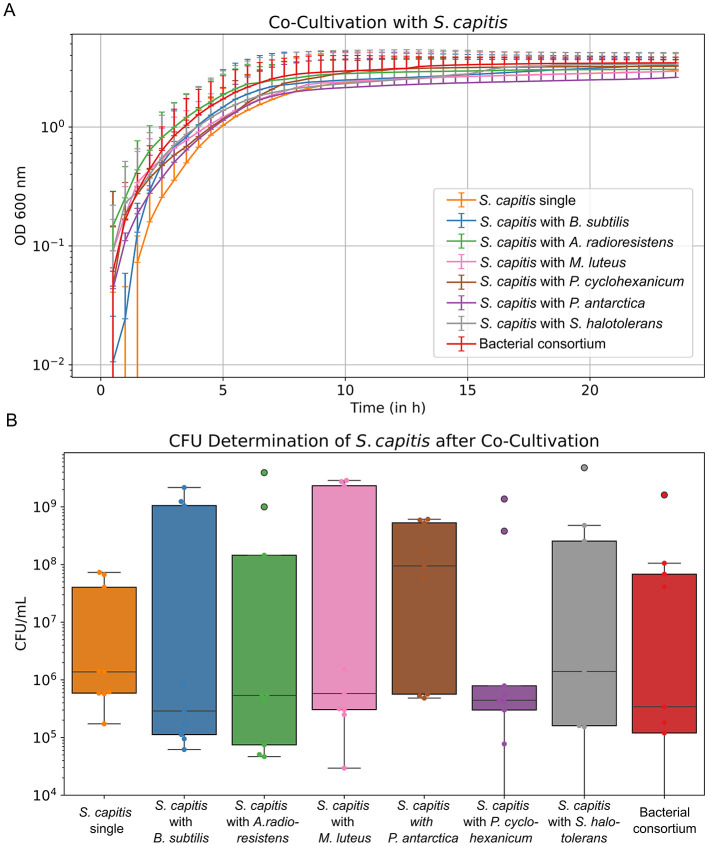
Co-cultivation approaches with *S. capitis*. **(A)** Growth curve of *S. capitis* as single species and in co-cultivation with each member of the bacterial consortium, respectively, and of the bacterial consortium. Pairwise co-cultivation with *S. capitis* is given as “with species name.” **(B)** Determination of CFU/mL of *S. capitis* after 24 h of co-cultivation in combination with each different species of the bacterial consortium and within the bacterial consortium. Error bars crossing the x-axis indicate no detectable culturable recovery for replicates of *P. cyclohexanicum* (*n* = 1), *S. halotolerans* (*n* = 1), and the bacterial consortium (*n* = 2).

Growth of *B. subtilis* was observed in all combinations, including the bacterial consortium and pairwise combinations with other consortium members. Growth of *B. subtilis* in co-cultivation with *P. antarctica* showed the lowest accumulation of biomass during the incubation period. Growth of *B. subtilis* and *S. halotolerans* showed a slower increase in biomass accumulation compared to other combinations (Figure 3A). Quantification of viable cells of *B. subtilis* revealed an increase of CFU/mL when co-cultivated with *A. radioresistens* and *M. luteus*, respectively, and decrease of CFU/mL when co-cultivated with *S. capitis* and *P. antarctica*, and a statistically significant decrease of CFU/mL when co-cultivated with the whole consortium (*p* = 0.008) (Figure 3B). But for one replicate each of pairwise combination with *S. capitis* and of the bacterial consortium, there was no detectable culturable recovery under the tested conditions of *B. subtilis*.

Growth was observed for all co-cultivation combinations with *S. capitis*, and with the lowest overall accumulation of biomass when co-cultivated with *P. antarctica*. When cultivated as single species, *S. capitis* showed the slowest increase of biomass accumulation in the exponential growth phase (Figure 4A). Determination of CFU/mL of *S. capitis* after co-cultivation showed an overall high variation, and no statistically significant difference compared to CFU/mL values of *S. capitis* when cultivated as single species (Figure 4B). However, in co-cultivation with *P. cyclohexanicum, S. halotolerans* (*n* = 1 each) as well as with the bacterial consortium (*n* = 2), replicates with no detectable culturable recovery of *S. capitis* were retrieved.

#### 3.2.1 Early-stage biofilm formation

Biofilm formation is a major strategy of microorganisms to resist and survive against harsh conditions. To analyze biofilm formation of *B. subtilis* and *S. capitis* as single species and of the whole bacterial consortium, we performed the semi-quantitative crystal violet assay (Figure 5, Supplementary Figure 3). While crystal violet solely measures biomass, it often serves as an indicator of biofilm formation. Hence, in the following biofilm formation refers to the measurement of biomass. Early-stage biofilm formation was highest for *A. radioresistens* and *B. subtilis*, followed by *M. luteus, P. antarctica* and *S. capitis*. The lowest biofilm formation was detected for *P. cyclohexanicum, S. halotolerans* and the bacterial consortium. Biofilm formation of *B. subtilis* (*p* = 0.021) and *A. radioresistens* (*p* = 0.004) were significantly higher than biofilm formation of the bacterial consortium (Supplementary Figure 3).

**Figure 5:**
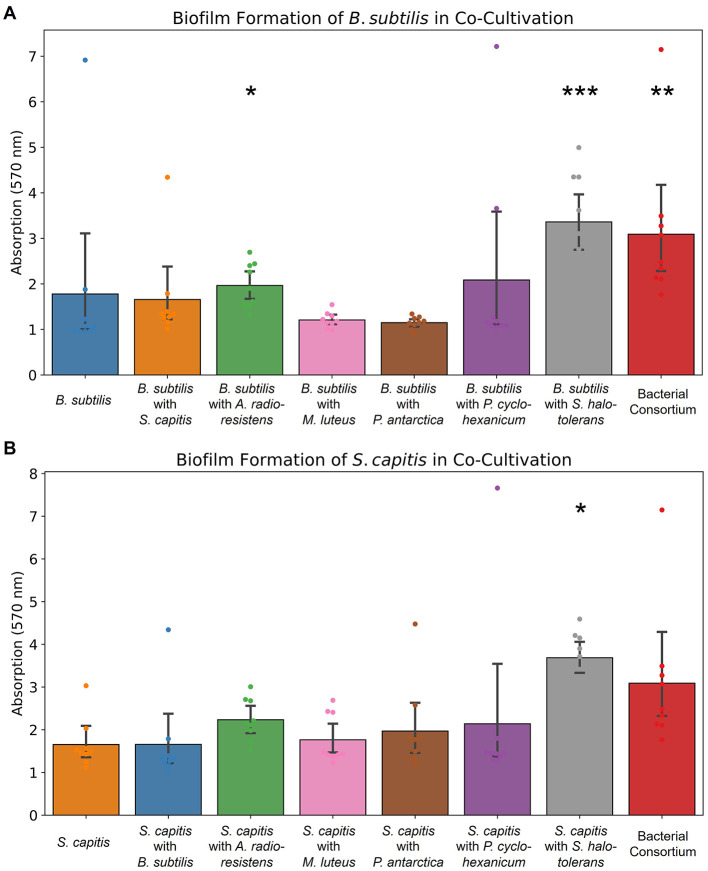
Surface attachment in co-cultivation of **(A)** *B. subtilis* and **(B)** *S. capitis*. The semi-quantitative crystal violet assay was performed to determine biofilm formation. The species were incubated statically at 37 °C for 24 h. Biofilm formation is proportional to absorption at 570 nm. The experiment was repeated independently three times with three biological replicates each. Statistical significance was tested between biofilm formation of the bacterial consortium, pairwise co-cultivation combinations with **(A)** *B. subtilis* and **(B)** *S. capitis*, respectively, and is indicated by asterisks (^*^p ≤ 0.05, ^**^p ≤ 0.005, ^***^p ≤ 0.001). Pairwise co-cultivation with *B. subtilis* **(A)** and *S. capitis* **(B)** is given as “with species name” correspondingly. The number of cells of *S. capitis* and *B. subtilis*, respectively, is equal in pairwise combinations and single cultivation, and thus the total number of cells in single cultivations of *S. capitis* and *B. subtilis*, respectively, is lower.

To investigate how the different species show potential synergistic, antagonistic or otherwise altered biofilm-formation capacities during early-stage biofilm formation and surface attachment, we performed crystal violet assays of co-cultivation approaches with *B. subtilis* and *S. capitis*, respectively, with the other species of the bacterial consortium (Figure 5). For determination of biofilm formation in pairwise combinations and *B. subtilis* and *S. capitis* as single species, the OD_600nm_ of the bacterial suspension of each respective species was adjusted to be equal. Conclusively, the initial OD_600nm_ and total number of cells in single species biofilm samples was lower.

Biofilm formation of *B. subtilis* in combination with *A. radioresistens* (*p* = 0.047), *S. halotolerans* (*p* < 0.001) and of the whole consortium (*p* = 0.002) was significantly higher compared to biofilm formation of *B. subtilis* as single species (Figure 5A). Biofilm formation of *S. capitis* was only significantly increased in pairwise combination with *S. halotolerans* (*p* = 0.002), but not in combination with other species of the bacterial consortium. Biofilm formation of the bacterial consortium was higher than biofilm formation of *S. capitis* as single species (Figure 5B).

### 3.3 Survival after co-exposure

The presence of other microorganisms can strongly influence survival and metabolism of single species. In order to test this, a straight-forward approach was started to determine how the presence of a synthetic bacterial consortium influences the survival of two species, namely *B. subtilis* and *S. capitis*, when co-exposed to different space-relevant stressors such as desiccation, X-ray irradiation, and oxidative stress.

#### 3.3.1 Desiccation

To determine desiccation tolerance, both *B. subtilis* and *S. capitis* were exposed to desiccation as single species and co-exposed within a bacterial consortium (Figure 6).

**Figure 6:**
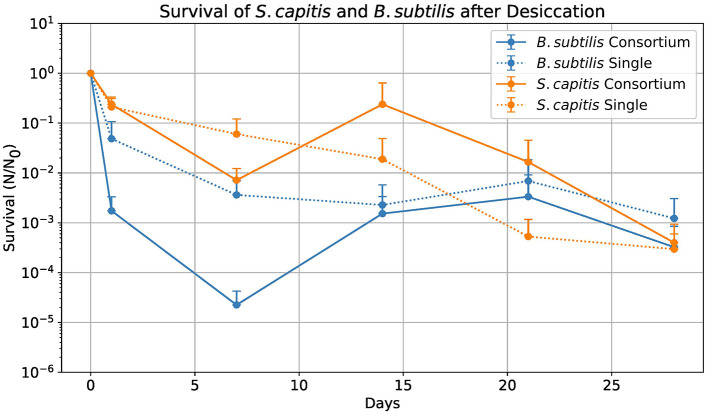
Survival fraction of *B. subtilis* and *S. capitis* exposed to desiccation as single species and co-exposed within the bacterial consortium. The desiccation tolerance was determined by dividing the exposed cell count after the respective days (N) by the initial cell count (N_0_). The average of three independent experiments with three biological replicates each is given.

Survival of *B. subtilis* as a single species decreased 2-log up to day 7 of desiccation, whereupon it remained relatively stable until day 28 with a 3-log reduction. In the bacterial consortium, survival of *B. subtilis* decreased more than 4-log up to day 7, but increased again until day 21, followed by a slight decline at day 28 with a 3-log reduction (Figure 6). Survival of *S. capitis* dropped until day 7. At day 14, survival of *S. capitis* in the bacterial consortium exceeded survival of *S. capitis* as single species. Beyond day 14, survival of *S. capitis* declined, with *S. capitis* as single species showing less survival than *S. capitis* within the bacterial consortium, but reached a similar level of a 3-log reduction on day 28 (Figure 6).

#### 3.3.2 X-ray irradiation

To determine X-ray irradiation tolerance, we exposed both species, *B. subtilis* and *S. capitis* as single species and within the bacterial consortium to cumulative doses ranging from 50 Gy to 500 Gy (Figure 7).

**Figure 7:**
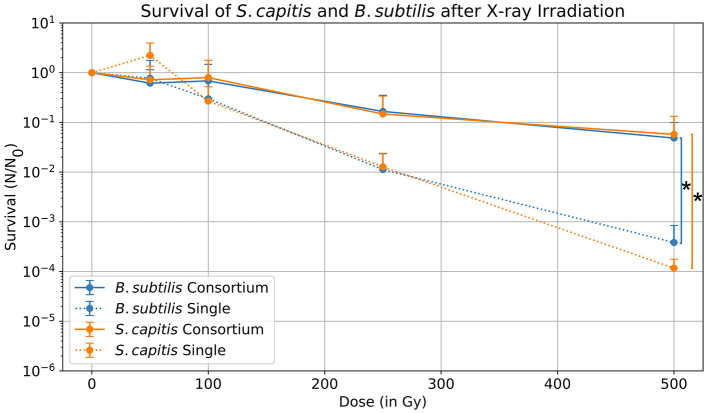
Survival fraction of *S. capitis* and *B. subtilis* after X-ray irradiation exposed as single species and co-exposed within the consortium. The tolerance toward X-ray irradiation was determined by dividing the cell count after exposure (N) by the initial cell count which was not exposed to X-ray irradiation (N_0_). Significance is indicated by an asterisk (^*^p ≤ 0.05).

Survival of *B. subtilis* and *S. capitis* decreased after X-ray irradiation with 500 Gy approximately 1-log for co-exposure within the bacterial consortium and 3 to 4-log after exposure to 500 Gy as single species. Survival of *B. subtilis* within the consortium was higher compared to exposure as single species (*p* = 0.012). Additionally, survival of *S. capitis* exposed as single species was overall higher after irradiation compared to *S. capitis* in the bacterial consortium and significantly increased after a cumulative dose of 500 Gy (*p* = 0.034).

#### 3.3.3 Hydrogen peroxide treatments

To determine hydrogen peroxide tolerance, we exposed both *B. subtilis* and *S. capitis* as single species and within the bacterial consortium to 1.5% and 3% hydrogen peroxide for 45 min and 90 min, respectively (Figure 8).

**Figure 8:**
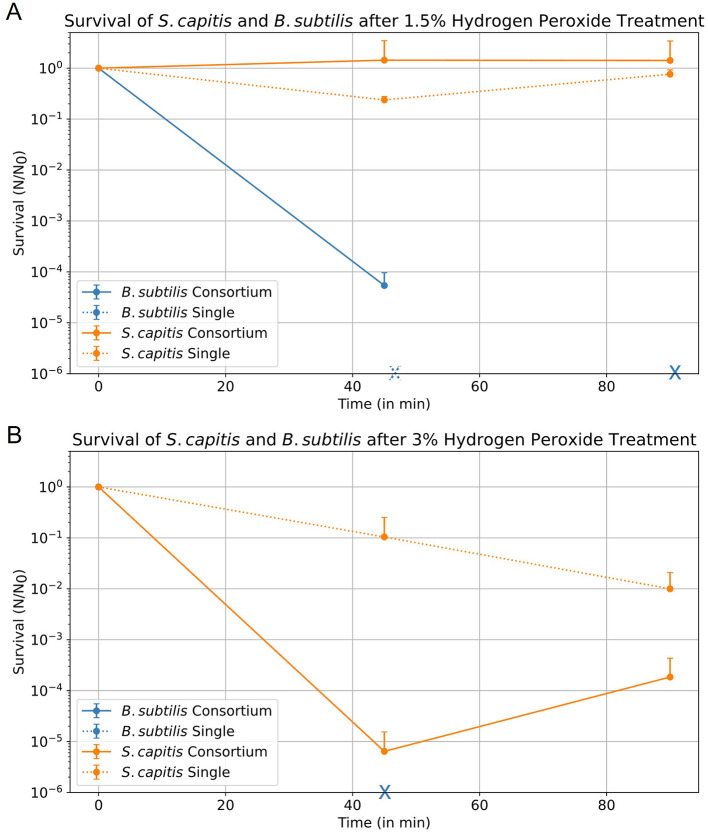
Tolerance toward **(A)** 1.5 % and **(B)** 3 % hydrogen peroxide exposure as single species and within bacterial consortium of *B. subtilis* and *S. capitis*. The survival fraction was determined by dividing the cell count after exposure to hydrogen peroxide (N) by the initial cell count (N_0_). Three independent experiments with three biological replicates each were performed. No detected survival is marked by an X.

Only vegetative cells of *B. subtilis* co-exposed within the bacterial consortium survived exposure to 1.5% hydrogen peroxide for 45 min with a minor survival fraction of less than 0.01% of the cells. We did not detect survival after exposure to other concentration and timepoint variations.

Both *S. capitis* exposed as single species and co-exposed within the bacterial consortium, showed less than a 1-log decrease in survival after treatment with 1.5% hydrogen peroxide for 45 min and 90 min. Survival of *S. capitis* co-exposed within the bacterial consortium, showed no decrease in survival after exposure to 1.5% for up to 90 min. Survival of *S. capitis* exposed as single species to 3% hydrogen peroxide, decreased steadily up to 2-log after 90 min. Survival of *S. capitis* co-exposed to 3% hydrogen peroxide, decreased by 5-log after 45 min but was higher (overall decrease by 4-log) after 90 min. This discrepancy is likely due to technical variability or a stochastic recovery artifact.

### 3.4 Consortium dynamics after co-cultivation and co-exposure

To identify changes in the consortium's relative abundance after co-cultivation and after stress exposure, 16S rRNA gene sequencing was performed as a first pre-test (Figure 9). Stress interventions included, analogous to the co-exposure experiments, desiccation, X-ray irradiation, and hydrogen peroxide exposure. 16S rRNA gene sequencing functioned primarily as a pretest or pilot investigation and was limited by an insufficient sample size. Additionally, it has to be noted that 16S rRNA gene sequencing generates compositional data rather than absolute abundance measurements.

**Figure 9:**
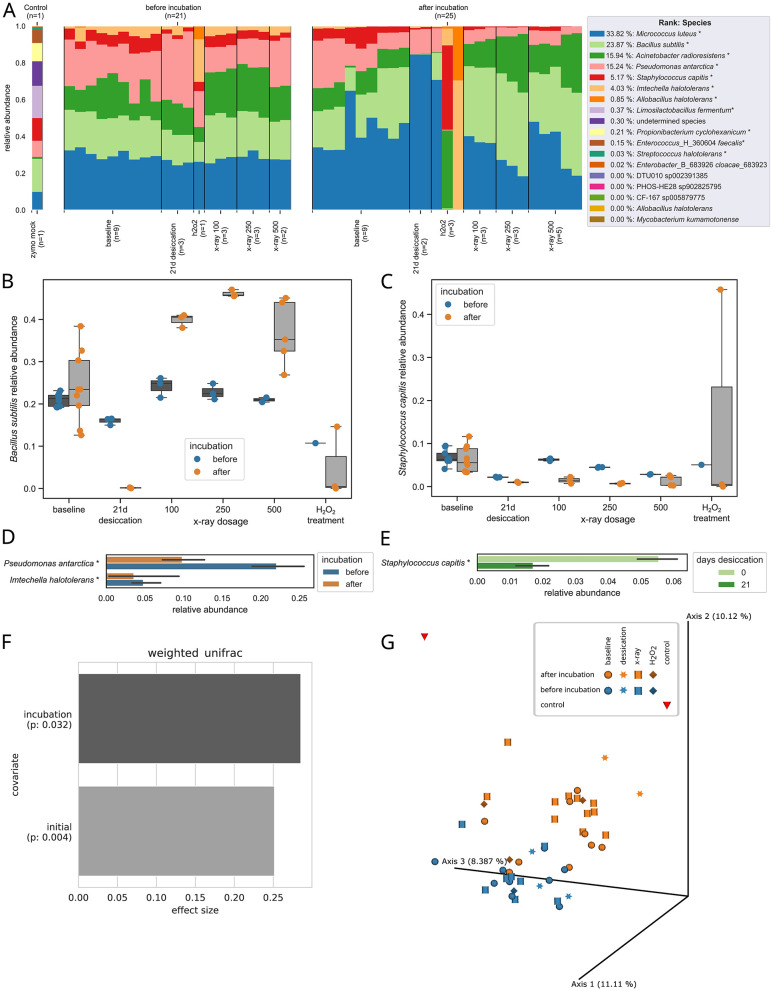
Consortium Dynamics: **(A)** Taxonomic composition sorted by before and after incubation on species level. Asterisks indicate species of the bacterial consortium, ZymoBIOMICS Spike-In control or ZymoBIOMICS microbial standard community. **(B, C)** Relative abundances of *B. subtilis* and *S. capitis* sorted by treatment and incubation timepoint. Baseline samples symbolize samples not exposed to stress treatments. **(D, E)** Mean relative abundances of ANCOM results showing differentially abundant samples between incubation timepoints and desiccation groups, respectively. **(F)** Effect sizes based on weighted UniFrac dissimilarity. A forward step redundancy analysis with a linear model was used including the parameters days of desiccation, experiment type, hydrogen peroxide (h2o2) treatment, incubation timepoint and overall stress (no stress controls). **(G)** 3D visualization of a PCoA of unweighted UniFrac. Colors adhere to incubation timepoint whereas shapes correlate with stress treatment.

A clear difference was observed between the consortium composition when assembled (before incubation) and after co-cultivation (after incubation) for 18 h. Relative abundances shifted during incubation, resulting in a distinct consortium structure (Figure 9A). Incubation exerted the largest effect size within the consortium as also visible in the emperor plot, which shows distinct clustering of cultivated and uncultivated samples (Figure 9). No taxa fell below the detection threshold after co-cultivation, co-exposure or co-exposure followed by co-cultivation.

The three stressors exhibited a significant effect on the abundance of the taxa, both in the assembled consortium (before incubation) and in the co-cultivated consortium (after incubation). However, no significant differences were observed in the diversity measures following stress exposure.

Incubation of the consortium did not affect the abundance of *B. subtilis* (Figure 9B) or *S. capitis* under baseline conditions (Figure 9C). However, the abundance of *B. subtilis* decreased following desiccation stress and subsequent co-cultivation. In contrast, *B. subtilis* abundance increased after X-ray stress and co-cultivation. After hydrogen peroxide treatment, abundance patterns of *B. subtilis* were variable following incubation (Figure 9B). Hydrogen peroxide exerted the strongest overall influence on the consortium, although the sample size was insufficient for definitive conclusions (Figure 9A).

The abundance of *S. capitis* declined after desiccation compared to the baseline condition, with an even greater reduction observed following desiccation stress and subsequent incubation (Figure 9C). ANCOM analysis confirmed this trend, indicating a decrease in *S. capitis* abundance by day 21 of desiccation (Figure 9E). When not co-cultivated (before co-cultivation), *S. capitis* abundance decreased with increasing X-ray dose; however, in the radiation-stressed, co-cultivated consortium, abundance remained stable (Figure 9C). Following hydrogen peroxide treatment, *S. capitis* abundance exceeded that of *B. subtilis*.

Cultivation most strongly affected *P. antarctica*, resulting in decreased abundance after co-cultivation. Similarly, *M. luteus* abundance declined following co-cultivation. The abundance of *Imtechella halotolerans* and *Allobacillus halotolerans*, included in the added ZymoBIOMICS Spike-in Control, showed high relative abundance in hydrogen peroxide treated samples (Figure 9A). Relative abundance of *I. halotolerans* exhibited high variance in relative abundance (Figure 9D). The observed increase in the relative abundance of spike-in taxa was consistent with a substantial reduction in recoverable consortium-derived bacterial signal following hydrogen peroxide treatment, although these observations should be considered preliminary. Additionally, CFU-based survival assays further support this interpretation, particularly the pronounced loss of recoverable *B. subtilis* and decreased survival at higher hydrogen peroxide concentrations (Figure 8). However, the current data do not clarify whether these shifts result from absolute biomass reduction, DNA degradation, selective survival, or a combination of these factors. Definitive interpretation will require additional approaches, such as qPCR-based absolute quantification, validated spike-in normalization, or direct biomass or cell-count measurements.

## 4 Discussion

Humanity's renewed commitment to reaching the Moon and Mars raises a plethora of considerations of which the microbiome and microorganisms are essential factors that need to be considered. Once in space, the microorganisms that demonstrate resilience, among which are numerous specialists in survival and adaptation (Mora et al., 2016b), encounter the distinctive conditions exhibited in the various space environments. However, it is important to note that microorganisms are not exposed in isolation, but rather as part of the microbial consortium in which they reside. The objective of the present study was to assess the resistance of two bacterial species, *S. capitis* and *B. subtilis*, to space-related stressors within a synthetic bacterial consortium, and to compare their survival when exposed as a single species. The survival of both *S. capitis* and *B. subtilis* was not generally enhanced or diminished, but instead, the consortium context modified stress responses depending on the specific stressor. Additionally, we investigated the effect of co-cultivation on biofilm formation and CFU recovery of *B. subtilis* and *S. capitis*.

### 4.1 Rationale of consortium selection and testing

We decided to investigate resistance changes in *Bacillus* and *Staphylococcus* since they have been reported to be prevalent in ISS and Mir station samples (Schiwon et al., 2013; Novikova, 2004; Novikova et al., 2006). Additionally, studies showed that *Staphylococcus* and *Bacillus* have high potential for co-selection after exposure to disinfection procedures to antibiotics (Sheikh et al., 2025; Sekiguchi et al., 2004). Since our aim was to investigate resistance within a bacterial consortium, to test *B. subtilis* we used vegetative cells instead of spores to allow putative metabolic interaction during co-exposure. However, this led to overall lower tolerance to desiccation, X-ray irradiation and hydrogen peroxide compared to previous studies testing *B. subtilis* spores (Moeller et al., 2012; Horneck et al., 2010).

Another limitation of the study is the lack of distinction between cell death and entry into non-culturable dormant states. This distinction is important since *B. subtilis* vegetative cells were intentionally used, but non-spore-forming actinobacteria such as *M. luteus* are capable of entering viable but non-culturable (VBNC) or non-replicative persistent states (Mukamolova et al., 2006; Mali et al., 2017; Havis et al., 2019; Li et al., 2014; Tirumalai et al., 2025). Although *M. luteus* was not among the two species selected for detailed stress analyses, it represents a consortium member with documented dormancy-resuscitation mechanisms. Consequently, reduced CFU recovery corresponds to decreased cultivability rather than definitive loss of viability and loss of CFU recovery should not be interpreted as definitive loss of viability. Conducting additional viability or resuscitation assays could aid in deciphering between loss of viability or cultivability.

### 4.2 Co-cultivation of consortium

One limitation of this study is that incubation was conducted at 37 °C, a temperature that is not optimal for the growth of *P. antarctica*. Consortium dynamics also showed a decrease of *P. antarctica* after cultivation. This species was included as a surrogate for *P. aeruginosa*, which has demonstrated increased virulence and biofilm formation under simulated microgravity (Chen et al., 2024; Kim et al., 2013). The incubation conditions could have led to reduced influence of *P. antarctica* on the bacterial consortium during the co-cultivation approaches. Additionally, medium and other cultivation conditions could be further improved. Co-cultivation approaches at an incubation temperature of 20 °C to 23 °C would enable an even closer simulation of indoor space habitat conditions. In future research, changes in abundance may serve as indicators for potential improvements in cultivation conditions. However, overall, the co-cultivation enabled the consortium to transition from an assembled state to a self-stabilizing consortium.

The observed reduction in viable cells of *B. subtilis* after co-cultivation within the bacterial consortium may be attributable to additional stressors, including the potential synthesis of antimicrobial peptides or toxins by competing bacterial taxa as has been studied for microbial communities in soil (Romdhane et al., 2021; Tyc et al., 2014). The genomes of the space habitat consortium members harbored different gene clusters for putative production of secondary metabolites including antimicrobial peptides, which could influence survival after stress exposure as well as in co-cultivation. However, it has to be noted that these findings reflect genomic potential rather than confirmed metabolite activity. Consistent with literature we found gene clusters for ribosomal synthesized and post-translationally modified peptides (RiPPs), as well as different peptide antibiotics in the genome of *B. subtilis*. *B. subtilis* produces non-ribosomal peptides (NRPs) and RiPPs (Puan et al., 2023). Antimicrobial peptides produced by *B. subtilis* have been shown to be antibacterial against other *Bacillus* spp., *M. luteus, Streptococcus pyogenes, Staphylococcus epidermidis*, and *Staphylococcus aureus* (Chalasani et al., 2015; Sharma et al., 2020). In the genome of *S. capitis*, we found different gene clusters for NRPS as well as RiPP-like products. *S. capitis* is known to produce a variety of different antimicrobial peptides including micrococcin P1, capidermicin, and other lantibiotics and exhibited antibacterial activity against *M. luteus, S. aureus, S. pyogenes, Bacillus coagulans, S. epidermidis, Enterococcus faecalis*, and *Enterococcus facecium* (Ohdan et al., 2026; Szekat et al., 2023). While we did not identify potential gene clusters of *A. radioresistens* for the production of antimicrobial peptides, we did find potential gene clusters for siderophores and aryl polyene, which contribute to resilience and virulence (Lee et al., 2021; Sheldon and Skaar, 2020). *A. radioresistens* has so far mostly been studied for its environmental resilience and as a potential reservoir of antibiotic resistance genes. Other species of the *Acinetobacter* genus, such as *Acinetobacter baumannii* have been mostly studied for their susceptibility toward antimicrobial peptides due to its clinical relevance (Hosseini et al., 2025). We found gene clusters for the production of terpenes similar to carotenoids, as well as for non-alpha poly-amino acids in the genome of *M. luteus*. *M. luteus* and related species have been shown to exhibit antimicrobial potential which has been attributed in part to carotenoid pigments, and other bioactive compounds such as micrococcin P1, kocurin and triclosan (Tizabi and Hill, 2023). Additionally, crude extracts of an *M. luteus* isolate were found to be antibacterial toward several multidrug-resistant isolates including species of *Acinetobacter* sp., *Pseudomonas* sp., and methicillin-resistant *Staphylococcus* sp. (Nisha et al., 2020). In the genome of *S. halotolerans* gene clusters putatively producing RiPP-like products, and a polyketide synthase were identified. While literature does not report production of antimicrobial peptides in *S. halotolerans* specifically, other streptococcal RaS (radical S-adenosyl-L-methionine)-RiPPs, RiPP-like products have been shown to exhibit antimicrobial activity (Clark et al., 2022). While the genome of *P. cyclohexanicum* harbored gene clusters responsible for the production of NRPS, we found no evidence of *P. cyclohexanicum* producing antimicrobial peptides, but another *Propionibacterium* sp. such as *Propionibacterium jensenii* has shown to produce bacteriocins (Wang et al., 2014). The genome of *P. antarctica* contained gene clusters for putative production of siderophores, NRPS, and aryl polyene. One study found that *P. antarctica* exhibited antimicrobial activity, and its genome harbored bacteriocin-producing genes (Lee et al., 2017), and another study found *Pseudomonas* spp. capable of producing bacteriocin-like compounds (Calisto et al., 2024). However, other *Pseudomonas* sp., such as *P. aeruginosa*, are mostly investigated for their susceptibility toward AMPs (Chen et al., 2023). The large number and diversity of different biosynthetic gene clusters, also involved in the production of antimicrobial peptides, indicate that interactions within the consortium are likely highly complex and require more in-depth analysis. Interestingly, a study showed that under simulated space stressors biosynthesis of antibiotics increased (Lu et al., 2023).

### 4.3 Early signs of biofilm formation

Biofilm formation is a well-established microbial strategy to withstand environmental and host-associated stressors including phagocytic clearance by human macrophages (Hernández-Jiménez et al., 2013). However, because crystal violet only quantifies biomass without normalization per cell, and does not assess biofilm architecture or viability, these measurements serve only as indicators of biofilm formation. In this study, biofilm formation of *B. subtilis* and *A. radioresistens* were highest among the tested members as single species. This observation aligns with *B. subtilis* characterization as a robust and versatile biofilm former, capable of producing complex extracellular matrices that enhance survival under desiccation, nutrient limitation and other environmental pressures (Muratov et al., 2025).

*S. capitis* has also been established as a biofilm former, and exhibited similar biofilm formation as single species aligning with values reported in Siems et al. (2022), and was higher compared to biofilm formation of the bacterial consortium. Among the different members, biofilm formation of the bacterial consortium was among the lowest, which was unexpected, given that natural biofilms are frequently multi-species structures in which interspecies interactions promote matrix production, spatial organization, and collective resistance (Berlanga and Guerrero, 2016). However, it has to be considered that this bacterial consortium is a synthetic construct, predicated on detected taxa, and consequently, interspecies interactions within the consortium may not be optimal. Competitive interactions within the synthetic community may suppress biofilm-associated traits, or certain members may inhibit matrix formation through metabolic interference or quorum-sensing disruption (Sun et al., 2023). Alternatively, the conditions optimized for monoculture biofilm formation, including choice of medium and incubation conditions, may not support cooperative biofilm development in a mixed community. These findings highlight that multi-species biofilm formation is not inherently guaranteed and may depend strongly on the specific ecological relationships and environmental context of the consortium.

Interestingly, biofilm formation of both *S. capitis* and *B. subtilis* in co-cultivation was highest in pairwise combination with *S. halotolerans*. One potential explanation could be that due to cultivation bias, since *S. halotolerans* did not exhibit substantial growth at 37 °C, the other consortium species may have utilized *S. halotolerans* cells as a nutrient source, even though the initial cell number of *S. halotolerans* was low.

In co-cultivation, the biofilm of the bacterial consortium increased significantly compared to the biofilm formation of *B. subtilis* as a single species when the initial optical density of single species and consortium was equivalent. However, when the number of cells in the respective bacterial suspensions for *B. subtilis* was equivalent, biofilm formation of the consortium was significantly higher. These contrasting outcomes suggest that biofilm formation of the bacterial consortium is highly sensitive to changes. The divergent biofilm formation of the consortium could reflect emergent properties of the consortium, where interspecies interactions such as matrix production respond non-linearly to small changes in local conditions. Such outcomes might be less apparent in monocultures, which showed more consistent biofilm formation across assays in relation to the initial cell number. However, it is important to note that the assessment of biofilm formation using crystal violet does not give absolute values but is rather used as a semi-quantitative measure of biofilm formation. Testing biofilm formation over an extended time period combined with molecular-biological approaches to identify the abundance of the single species within could aid in gaining further insight into the biofilm formation of the consortium. Studies have shown that biofilm formation of consortia suggest interspecific co-operations, enhanced adaptation and increased resistance toward sanitizers (Henriksen et al., 2022; Pant et al., 2025; Ren et al., 2014). Additionally, biofilm formation has been linked to increased resistance (Giaouris et al., 2014). This would also be crucial for space applications since some studies have pointed toward increased biofilm formation in space isolates and under spaceflight conditions (Kim et al., 2013; Mora et al., 2019), which can negatively impact materials and astronaut health.

### 4.4 Desiccation tolerance

Microorganisms with high desiccation tolerance are another challenging factor relevant to planetary protection. Studying microbial desiccation resilience can help agencies refine sterilization protocols and prevent forward contamination during missions. The reduced desiccation survival of *B. subtilis* observed in this study likely reflects the use of vegetative *B. subtilis* cells rather than the highly desiccation-resistant spores described in literature. *B. subtilis* spores are able to resist desiccation due to their low water content, protective small acid-soluble proteins and robust DNA repair mechanisms (Moeller et al., 2014). Survival of *Bacillus* depends on sporulation and biofilm formation, and *B. subtilis* is part of the 500-year experiment demonstrating the spores' resistance (Branda et al., 2001; Dawes and Mandelstam, 1970; Ulrich et al., 2018; Wahlen et al., 2018). Sporulation and biofilm formation could be affected through diverse interactions within the bacterial consortium, explaining the low survival rate. However, after an initial decline in survival, after day 14 of desiccation, survival of *B. subtilis* increased within the bacterial consortium. This could point toward *B. subtilis* cells showing resilience, as in being able to recover after exposure to a stressor, and returning to an increased survival (Shade et al., 2012). One possible explanation could be that this may have been a technically introduced error or it could be due to the presence of spores in the bacterial suspension. Additionally, cannibalism has been shown to delay sporulation in *B. subtilis* cells, which could explain an initial decrease followed by an increase of survival (López et al., 2009).

Survival of *S. capitis* as single species toward desiccation matched values reported in literature (Siems et al., 2022). *S. capitis* co-exposed in the bacterial consortium showed an initial decrease in survival until day 7 of desiccation, followed by increased survival that was higher than the survival of *S. capitis* as single species. This enhanced survival of *S. capitis* within the bacterial consortium could be due to release of nutrients from species which are more desiccation-sensitive and hence, underwent lysis thereby offering more nutrients for the desiccation-tolerant species within the bacterial consortium. Additionally, dead cells of other bacteria could have served as a physical protective layer. Since the size of *S. capitis* is smaller than most of the size of the other members of the consortium, this could mean that it absorbs released nutrients more efficiently due to the higher surface to volume ratio (Young, 2006). Additionally, the fast growth physiology of *S. capitis* could result in consumption of released nutrients which increases survivability.

### 4.5 X-ray radiation resistance

Radiation exposure on the ISS is dependent on its altitude and solar activities (Berger et al., 2016) and astronauts on the ISS receive approximately 110-180 mSv radiation exposure, compared to approximately 2.4 mSv on Earth (Restier-Verlet et al., 2021). In space habitats, not only the crew and equipment are exposed to higher radiation, but also the present microorganisms. X-ray irradiation is a significant stress factor, already 5 Gy of cumulative dose is lethal for humans, but distinct microorganisms can withstand 200-fold doses of the human lethal dose (Cortesão et al., 2020; Runzheimer and Leuko, 2025; Moeller et al., 2014). Hence it poses a stress factor which may impose selective pressure toward higher X-ray irradiation and influence survival. *B. subtilis* spores depict a high survival toward different environmental stressors including X-ray irradiation with studies reporting mean D_10_ values of 857 Gy for *B. subtilis* spores (Moeller et al., 2014) compared to 152 Gy in this study exposed as single species. The lower observed X-ray irradiation tolerance of *B. subtilis* in this study is attributed to the use of vegetative cells. The resistance of *B. subtilis* spores relies on multiple protective strategies as described above for desiccation tolerance. However, vegetative cells do not possess these strategies. Additionally, different phenotypes and genotypes of *B. subtilis* strains were found to vary in X-ray irradiation resistance (Moeller et al., 2014). Survival of *S. capitis* exposed as single species aligns with literature values (~98 Gy) reported in Siems et al. (2022), even though the determined LD_90_ values in this study were higher (155 Gy), which could be due to experimental differences such as starting OD_600nm_.

Remarkably, survival of both *B. subtilis* and *S. capitis* within the bacterial consortium was increased with a more than doubled LD_90_ of 370 Gy (*B. subtilis*) and 384 Gy (*S. capitis*) compared to the single species results. For both species, survival after co-exposure within the bacterial consortium was 3-log higher after exposure to 500 Gy and significantly increased compared to survival after exposure as single species. This finding suggests that the bacterial consortium provides protective effects to both species or consortium interactions positively affected survival. Another study showed that the stress resistance of bacterial consortia relied on the ability of the individual members to adapt their metabolism and gene expression, enhancing mutual protection (Shade et al., 2012).

### 4.6 Hydrogen peroxide tolerance

Since the ISS is a unique habitat, cleaning needs to be adapted to these unique conditions. Cleaning on the ISS is performed using hydrogen peroxide wipes (Vijapur et al., 2022; Viroxtechnologies, 2018), as they are not only good for cleaning, but also safe in handling, since their chemical end products are oxygen and water. When exposed to hydrogen peroxide as single species, vegetative cells of *B. subtilis* did not survive any treatment, but *B. subtilis* co-exposed within the consortium showed survival after treatment with 1.5% of hydrogen peroxide for 45 min. The overall low tolerance of vegetative cells of *B. subtilis* toward hydrogen peroxide has also been shown in another publication, where tolerance to hydrogen peroxide was attributed to which catalase was used (Handtke et al., 2017). Another study showed that the biofilm matrix built by *B. subtilis* protected its cells from hydrogen peroxide (Muratov et al., 2025). Pre-tests revealed low survival fraction of vegetative cells of *B. subtilis* already after 5 min treatment. For direct comparability of results, it was decided to use established testing points of *S. capitis* for both species. Consortium dynamics further indicated that *B. subtilis* abundance decreased more substantially than *S. capitis* following hydrogen peroxide exposure. This observation aligns with the co-exposure survival data.

Hydrogen peroxide tolerance of *S. capitis* showed similar trends as reported in literature, even though different timepoints were investigated (Siems et al., 2022). Survival of *S. capitis* after exposure to 3% hydrogen peroxide was higher when exposed as single species compared to co-exposure within the consortium. Decreased survival of *S. capitis* co-exposed within the bacterial consortium might be due to the differential use of catalases among members of the consortium. The use of catalases, which convert hydrogen peroxide to oxygen and water molecules, is a major survival strategy against oxidative stress. Additionally, decreased survival of *S. capitis* after co-exposure within the bacterial consortium could be due to a competition for available nutrients between the different species within the consortium. Interestingly, after an initial decrease in survival of *S. capitis* after co-exposure to 3% hydrogen peroxide, survival increased again, possibly due to other less hydrogen peroxide tolerant species of the bacterial consortium being killed off and *S. capitis* being able to benefit from the released nutrients.

The different survival responses when exposed within a bacterial consortium indicate that survival of species within a consortium is highly dependent on the respective environmental stressor. Another study looking at the survival after exposure to copper surfaces found that the survival of *S. capitis* was higher when co-exposed within a consortium compared to exposure as single species (Ly-Sauerbrey et al., 2025).

### 4.7 Consortium dynamics

The abundance of *B. subtilis* decreased after desiccation stress, but remained relatively stable after X-ray irradiation before co-cultivation, and even increased after X-ray irradiation after co-cultivation. Another study found that in conditions of low-dose radiation and microgravity, the abundance of Bacilli increased, while the abundances of other species changed differently when exposed to a combination of stressors compared to single stressors (Lu et al., 2023).

In summary, analysis of consortium dynamics demonstrated a transition from the synthetically assembled consortium to a more natural, equilibrated state. The absence of taxa loss following stress exposure or co-cultivation indicates that the consortium maintains stability in both conditions suggesting that this approach was an effective strategy for combining these taxa into a functional consortium. Through investigation of abundance changes of bacterial consortium members, we confirmed trends detected in co-exposure experiments.

The elevated relative abundance of spike-in taxa following hydrogen peroxide treatment indicates a substantial reduction in consortium-derived bacterial signal. However, because 16S rRNA gene sequencing data are compositional, these findings require cautious interpretation. Shifts in relative abundance alone do not demonstrate absolute biomass loss, DNA degradation, or selective killing. To differentiate among these scenarios, absolute quantification methods such as qPCR, validated spike-in normalization, direct biomass measurements, or direct cell counting are necessary.

However, it should be noted that, within the scope of this study, consortium dynamics were assessed only as a preliminary test, and a greater number of replicates is required to strengthen these findings. Further research should include additional incubation periods and temperature conditions to validate these observations. Future studies may also employ additional genetic markers to improve species identification.

### 4.8 Implications and future research directions

While this study has given first insights into stress resistance potential within a synthetic space habitat bacterial consortium, there are many different ways in continuing and deepening this research. A first approach could involve conducting pairwise co-exposure experiments to complement co-cultivation studies and to determine whether specific species of the consortium are responsible for the changes in survival of *B. subtilis* and *S. capitis* observed during co-exposure experiments. Additionally, applying more refined tools could facilitate dissecting interactions within the bacterial consortium. Fluorescent labeling would enable visualization of spatial organization, dynamic behavior during co-cultivation, offering insight into long term distribution and interaction of individual species. Additionally, varying the ratios of the different members of the bacterial consortium could further deepen understanding how consortium composition influences stress responses. On a different level, complementing these approaches, quantitative molecular assays such as qPCR or transcriptomic analysis would enable analysis of consortium dynamics in gene-expression changes, cooperative or competitive interactions. Additionally, metabolomics would allow a more detailed analysis in metabolic exchange and resource utilization and their influence on stress tolerance in bacterial consortia. Together, these strategies could provide a more comprehensive understanding of mechanisms which shape bacterial stress resistance and resilience. Another aspect which could be researched is intercellular communication within the consortium, namely quorum sensing (Bassler and Losick, 2006; Kareb and Aïder, 2020). Quorum sensing affects various functions included in stress tolerance such as biofilm formation, virulence, sporulation, horizontal gene transfer, and biosynthesis of antimicrobial peptides (Kleerebezem, 2004; Bassler and Losick, 2006). *B. subtilis* is able to degrade acyl-homoserine lactones (AHL), which are quorum sensing signal molecules in gram-negative bacteria. This ability is facilitated by an AHL-lactonase (*aiiA*), thereby enhancing its competitiveness within a heterogenous bacterial population (Noor et al., 2022). While we did not find *aiiA* in the genome data of *B. subtilis*, we did find another lactonase putatively involved in quorum sensing. The genome of *S. capitis* harbored a gene cluster putatively responsible for the production of a quorum sensing signaling molecule, namely a cyclic-lactone autoinducer. Additionally, *agrA, agrC*, and *agrD* were found in the genome of *S. capitis*. In *S. capitis* quorum sensing has been shown to depend largely on an accessory gene regulator (Agr), which is activated by autoinducing peptides and involved in increased expression of toxins and degradative exoenzymes under high cell density, and biofilm formation (Le and Otto, 2015; Crepin et al., 2024; Novick and Muir, 1999).

Furthermore, studying consortium dynamics in space experiments and clarifying the effects of actual space conditions on these consortia will improve our understanding of interactions among commonly detected taxa in space habitats. Using synthetic bacterial consortia for research on space habitat management may aid in developing strategies to predict bacterial stress response and act as controllable model ecosystems to further understand bacterial interactions, stability and functions. This is crucial in space habitats, where microbial behavior can vary due to altered gravity, increased radiation exposure, reduced nutrient-availability, and confinement. Synthetic ecological frameworks could help in maintaining a stable microbiome in built habitats from which occupying humans could benefit. Additionally, microbial consortia may aid in vital processes such as bioregenerative life support and sustainable space exploration (Santomartino et al., 2023).

The resistance of specific species within a bacterial consortium may depend more on the combination of consortium context and the nature of the stressor than on the mere presence of the consortium. While growth was observable within the different co-cultivation conditions, more in-depth analysis is needed to reveal interspecies interactions. While there is endless potential for testing microbial consortia and their influence on resistance to certain stressors and environmental conditions, and the role of individual species, our study has established the basis for future work, allowing for a more realistic understanding of how bacterial interactions might affect tolerance toward space-related stressors.

## Data Availability

The 16S rRNA gene sequencing datasets generated for this study can be found in the European Nucleotide Archive (ENA) with the accession PRJEB112301 (ERP192879). The whole genome dataset of *B. subtilis* W2-B1-K4 generated for this study has been deposited at DDBJ/ENA/GenBank under the accession JBYFEL000000000. The version described in this paper is version JBYFEL010000000. The remaining datasets analyzed for this study can be found in NCBI with the INSDC accessions given in Table 1.
