# Supplementary material for: Community matters: stress tolerance and survival of Bacillus subtilis and Staphylococcus capitis in a synthetic space habitat consortium
Source: Front Microbiol. 2026 Jul 27;17:1869903. doi: 10.3389/fmicb.2026.1869903 (PMC13454065; doi:10.3389/fmicb.2026.1869903)
Supplement: Supplementary file 1 [file Data_Sheet_1.docx]

Supplementary Material

# 1 Supplementary Data

## 1.1 Supplementary Tables

Supplementary Table 1: Antibiotic resistance testing of bacterial consortium. Antibiotic resistance of species of bacterial consortium were tested for later evaluation of growth of *S. capitis* and *B. subtilis*. To determine CFU/mL of *B. subtilis* streptomycin was added to agar plates, and rifampicin was added to agar plates for determination of CFU/mL of *S. capitis*.

| Bacterial Species | Rifampicin (in µg/mL) | | Streptomycin  (in µg/mL) | | | Erythromycin (in µg/mL) | | | Penicillin G  (in µg/mL) | | |
| --- | --- | --- | --- | --- | --- | --- | --- | --- | --- | --- | --- |
|  | 3 | 5 | 5 | 20 | 50 | 5 | 20 | 50 | 5 | 20 | 50 |
| *B. subtilis* | S | S | R | R | R | S | S | S | R | R | R |
| *S. capitis* | R | R | I | I | I | I | I | I | I | I | I |
| *A. radioresistens* | S | S | I | S | S | S | S | S | I | S | S |
| *M. luteus* | I | I | I | I | I | I | I | I | S | S | S |
| *P. antarctica* | S | S | I | I | I | I | I | I | R | R | R |
| *P. cyclohexanicum* | I | I | I | I | I | I | I | I | S | S | S |
| *S. halotolerans* | I | I | I | I | I | I | I | I | I | I | I |

Supplementary Table 2: Secondary metabolite biosynthesis gene clusters in genome data of consortium species using antiSMASH. For each species the product type of secondary metabolite is given in respect to the region in whole genome data, and the closest known compound from the MiBIG database with its respective type and corresponding similarity is given. Data was obtained in Galaxy using antiSMASH. Data is accessible on Galaxy by following the links of region and rost-similar known cluster. NRP= Non-ribosomal peptide, NRPS = Non-ribosomal peptide synthetase, PKS= Polyketide synthase, CDPS= tRNA-dependent cyclodipeptide synthases, RiPP= Ribosomally synthesized and post-translationally modified peptides, RRE= RiPP recognition element, NAPAA= Non-alpha poly-amino acids, LAP= Linear azol(in)e-containing peptides, NAGGN= N-acetylglutaminylglutamine amide, RaS-RiPP= Streptide-like thioether-bond RiPPs.

| *B. subtilis* | | | | | | |
| --- | --- | --- | --- | --- | --- | --- |
| **Region** | **Type** | **From** | **To** | **Most similar known cluster** | | **Similarity** |
| [Region 1](https://usegalaxy.org/datasets/f9cad7b01a472135b6d42e46f5fd1233/display/?preview=True#r1c1) | Ranthipeptide, Sactipeptide | 203,774 | 225,847 | [Sporulation killing factor](https://mibig.secondarymetabolites.org/go/BGC0000601/1) | RiPP:Head-to-tailcyclized peptide | 100% |
| [Region 2](https://usegalaxy.org/datasets/f9cad7b01a472135b6d42e46f5fd1233/display/?preview=True#r1c2) |  | 357,902 | 421,342 | [Surfactin](https://mibig.secondarymetabolites.org/go/BGC0000433/1) | NRP:Lipopeptide | 82% |
| [Region 3](https://usegalaxy.org/datasets/f9cad7b01a472135b6d42e46f5fd1233/display/?preview=True#r1c3) | Terpene | 1,149,540 | 1,170,343 |  | | |
| [Region 4](https://usegalaxy.org/datasets/f9cad7b01a472135b6d42e46f5fd1233/display/?preview=True#r1c4) | Trans-AT PKS, PKS-like, Type III PKS, NRPS | 1,766,685 | 1,863,293 | [Bacillaene](https://mibig.secondarymetabolites.org/go/BGC0001089/1) | Polyketide + NRP | 100% |
| [Region 5](https://usegalaxy.org/datasets/f9cad7b01a472135b6d42e46f5fd1233/display/?preview=True#r1c5) | Beta-lactone containing protease inhibitor | 1,944,954 | 1,969,097 | [Fengycin](https://mibig.secondarymetabolites.org/go/BGC0001095/1) | NRP | 53% |
| [Region 6](https://usegalaxy.org/datasets/f9cad7b01a472135b6d42e46f5fd1233/display/?preview=True#r1c6) | Glycocin | 2,109,980 | 2,130,150 | [Sublancin 168](https://mibig.secondarymetabolites.org/go/BGC0000558/1) | RiPP:Lanthipeptide | 100% |
| [Region 7](https://usegalaxy.org/datasets/f9cad7b01a472135b6d42e46f5fd1233/display/?preview=True#r1c7) | Type III PKS | 2,147,415 | 2,188,512 |  | | |
| [Region 8](https://usegalaxy.org/datasets/f9cad7b01a472135b6d42e46f5fd1233/display/?preview=True#r1c8) | NRPS | 3,110,976 | 3,158,112 | [Bacillibactin](https://mibig.secondarymetabolites.org/go/BGC0000309/1) | NRP | 100% |
| [Region 9](https://usegalaxy.org/datasets/f9cad7b01a472135b6d42e46f5fd1233/display/?preview=True#r1c9) | CDPS | 3,444,278 | 3,465,024 |  | | |
| [Region 10](https://usegalaxy.org/datasets/f9cad7b01a472135b6d42e46f5fd1233/display/?preview=True#r1c10) | Sactipeptide | 3,676,516 | 3,698,127 | [Subtilosin A](https://mibig.secondarymetabolites.org/go/BGC0000602/1) | RiPP:Thiopeptide | 100% |
| [Region 11](https://usegalaxy.org/datasets/f9cad7b01a472135b6d42e46f5fd1233/display/?preview=True#r1c11) | [Other](https://docs.antismash.secondarymetabolites.org/glossary/#other) | 3,701,126 | 3,742,544 | [Bacilysin](https://mibig.secondarymetabolites.org/go/BGC0001184/1) | Other | 100% |
| [Region 12](https://usegalaxy.org/datasets/f9cad7b01a472135b6d42e46f5fd1233/display/?preview=True#r1c12) | RRE-element containing cluster | 3,938,603 | 3,958,872 |  | | |
| [Region 13](https://usegalaxy.org/datasets/f9cad7b01a472135b6d42e46f5fd1233/display/?preview=True#r1c13) | Epipeptide | 3,966,195 | 3,987,893 | [Thailanstatin A](https://mibig.secondarymetabolites.org/go/BGC0001114/1) | NRP + Polyketide | 10% |
|  |  |  |  |  |  |  |
| *S. capitis* | | | | | | |
| **Region** | **Type** | **From** | **To** | **Most similar known cluster** | | **Similarity** |
| [Region 1.1](https://usegalaxy.org/datasets/f9cad7b01a4721352d74893d886a3e44/display/?preview=True#r1c1) | Glycosylated lanthipeptide; NRPS | 31,450 | 119,747 |  | | |
| [Region 1.2](https://usegalaxy.org/datasets/f9cad7b01a4721352d74893d886a3e44/display/?preview=True#r1c2) | Terpene, Type III PKS | 337,833 | 388,145 |  | | |
| [Region 1.3](https://usegalaxy.org/datasets/f9cad7b01a4721352d74893d886a3e44/display/?preview=True#r1c3) | Siderophore | 695,249 | 710,228 | [Staphyloferrin A](https://mibig.secondarymetabolites.org/go/BGC0000944/1) | Other:Non-NRP siderophore | 100% |
| [Region 1.4](https://usegalaxy.org/datasets/f9cad7b01a4721352d74893d886a3e44/display/?preview=True#r1c4) | Cyclic-lactone-autoinducer | 836,128 | 856,837 | [Kijanimicin](https://mibig.secondarymetabolites.org/go/BGC0000082/1) | Polyketide | 4% |
| [Region 1.5](https://usegalaxy.org/datasets/f9cad7b01a4721352d74893d886a3e44/display/?preview=True#r1c5) | Lanthipeptide | 1,740,831 | 1,765,049 | [Gallidermin](https://mibig.secondarymetabolites.org/go/BGC0000514/1) | RiPP:Lanthipeptide | 36% |
| [Region 1.6](https://usegalaxy.org/datasets/f9cad7b01a4721352d74893d886a3e44/display/?preview=True#r1c6) | RiPP-like | 1,889,709 | 1,900,101 |  | | |
| [Region 15.1](https://usegalaxy.org/datasets/f9cad7b01a4721352d74893d886a3e44/display/?preview=True#r15c1) | [Lanthipeptide](https://docs.antismash.secondarymetabolites.org/glossary/#lanthipeptide-class-i) | 1 | 5,730 | [Gallidermin](https://mibig.secondarymetabolites.org/go/BGC0000514/1) | RiPP:Lanthipeptide | 27% |
| [Region 48.1](https://usegalaxy.org/datasets/f9cad7b01a4721352d74893d886a3e44/display/?preview=True#r48c1) | Cyclic-lactone-autoinducer | 1 | 1,687 |  | | |
| [Region 73.1](https://usegalaxy.org/datasets/f9cad7b01a4721352d74893d886a3e44/display/?preview=True#r73c1) | RRE-containing | 1 | 1,097 |  | | |
|  |  |  |  |  |  |  |
| *M. luteus* | | | | | | |
| **Region** | **Type** | **From** | **To** | **Most similar known cluster** | | **Similarity** |
| [Region 1.1](https://usegalaxy.org/datasets/f9cad7b01a4721356890b75e6b090480/display/?preview=True#r1c1) | Ectoine | 242,693 | 252,875 |  | | |
| [Region 1.2](https://usegalaxy.org/datasets/f9cad7b01a4721356890b75e6b090480/display/?preview=True#r1c2) | RRE-containing | 555,817 | 576,149 |  | | |
| [Region 1.3](https://usegalaxy.org/datasets/f9cad7b01a4721356890b75e6b090480/display/?preview=True#r1c3) | Siderophore | 1,111,905 | 1,123,740 |  | | |
| [Region 1.4](https://usegalaxy.org/datasets/f9cad7b01a4721356890b75e6b090480/display/?preview=True#r1c4) | Beta-lactone containing protease inhibitor | 1,474,487 | 1,501,450 | [Microansamycin](https://mibig.secondarymetabolites.org/go/BGC0001666/1) | Polyketide | 7% |
| [Region 1.5](https://usegalaxy.org/datasets/f9cad7b01a4721356890b75e6b090480/display/?preview=True#r1c5) | NAPAA | 1,543,608 | 1,577,615 | [Stenothricin](https://mibig.secondarymetabolites.org/go/BGC0000431/1) | NRP:Cyclic depsipeptide | 31% |
| [Region 1.6](https://usegalaxy.org/datasets/f9cad7b01a4721356890b75e6b090480/display/?preview=True#r1c6) | Terpene | 2,328,008 | 2,348,520 | [Carotenoid](https://mibig.secondarymetabolites.org/go/BGC0000636/1) | Terpene | 21% |
|  |  |  |  |  |  |  |
| *P. antarctica* | | | | | | |
| **Region** | **Type** | **From** | **To** | **Most similar known cluster** | | **Similarity** |
| [Region 1.1](https://usegalaxy.org/datasets/f9cad7b01a47213509afede46f7253f4/display/?preview=True#r1c1) | NRPS-like | 111,945 | 154,826 | [Ambactin](https://mibig.secondarymetabolites.org/go/BGC0001131/1) | NRP | 25% |
| [Region 1.2](https://usegalaxy.org/datasets/f9cad7b01a47213509afede46f7253f4/display/?preview=True#r1c2) | Arylpolyene | 460,158 | 503,733 | [APE Vf](https://mibig.secondarymetabolites.org/go/BGC0000837/1) | Other | 40% |
| [Region 1.3](https://usegalaxy.org/datasets/f9cad7b01a47213509afede46f7253f4/display/?preview=True#r1c3) | RiPP-like | 1,487,121 | 1,495,834 |  | | |
| [Region 1.4](https://usegalaxy.org/datasets/f9cad7b01a47213509afede46f7253f4/display/?preview=True#r1c4) | NRPS, Terpene | 2,718,875 | 2,782,701 | [Pyoverdin](https://mibig.secondarymetabolites.org/go/BGC0000413/1) | NRP | 11% |
| [Region 1.5](https://usegalaxy.org/datasets/f9cad7b01a47213509afede46f7253f4/display/?preview=True#r1c5) | [NRPS](https://docs.antismash.secondarymetabolites.org/glossary/#nrps) | 3,595,824 | 3,658,358 | [Viscosin](https://mibig.secondarymetabolites.org/go/BGC0001312/1) | NRP | 75% |
| [Region 1.6](https://usegalaxy.org/datasets/f9cad7b01a47213509afede46f7253f4/display/?preview=True#r1c6) | [Beta-lactone containing protease inhibitor](https://docs.antismash.secondarymetabolites.org/glossary/#betalactone) | 3,823,656 | 3,843,639 | [Fengycin](https://mibig.secondarymetabolites.org/go/BGC0001095/1) | NRP | 13% |
| [Region 1.7](https://usegalaxy.org/datasets/f9cad7b01a47213509afede46f7253f4/display/?preview=True#r1c7) | NRPS, LAP | 3,959,223 | 4,003,843 | [Tolaasin I / Tolaasin F](https://mibig.secondarymetabolites.org/go/BGC0000447/1) | NRP:Lipopeptide | 60% |
| [Region 1.8](https://usegalaxy.org/datasets/f9cad7b01a47213509afede46f7253f4/display/?preview=True#r1c8) | [NAGGN](https://docs.antismash.secondarymetabolites.org/glossary/#naggn) | 4,287,549 | 4,302,391 |  | | |
| [Region 1.9](https://usegalaxy.org/datasets/f9cad7b01a47213509afede46f7253f4/display/?preview=True#r1c9) | [NRPS](https://docs.antismash.secondarymetabolites.org/glossary/#nrps) | 4,335,699 | 4,388,595 | [Pyoverdin](https://mibig.secondarymetabolites.org/go/BGC0000413/1) | NRP | 9% |
| [Region 1.10](https://usegalaxy.org/datasets/f9cad7b01a47213509afede46f7253f4/display/?preview=True#r1c10) | Siderophore | 4,480,220 | 4,492,145 |  | | |
| [Region 1.11](https://usegalaxy.org/datasets/f9cad7b01a47213509afede46f7253f4/display/?preview=True#r1c11) | Beta-lactone containing protease inhibitor | 4,911,232 | 4,942,504 |  | | |
| [Region 1.12](https://usegalaxy.org/datasets/f9cad7b01a47213509afede46f7253f4/display/?preview=True#r1c12) | Redox-cofactor | 5,761,020 | 5,783,167 | [Lankacidin C](https://mibig.secondarymetabolites.org/go/BGC0001100/1) | NRP + Polyketide | 13% |
| [Region 1.13](https://usegalaxy.org/datasets/f9cad7b01a47213509afede46f7253f4/display/?preview=True#r1c13) | RiPP-like | 6,318,277 | 6,329,122 |  | | |
|  |  |  |  |  |  |  |
| *P. cyclohexanicum* | | | | | | |
| **Region** | **Type** | **From** | **To** | **Most similar known cluster** | | **Similarity** |
| [Region 1](https://usegalaxy.org/datasets/f9cad7b01a472135efd7fc12c4925d31/display/?preview=True#r1c1) | NRPS | 586,681 | 639,051 |  | | |
| [Region 2](https://usegalaxy.org/datasets/f9cad7b01a472135efd7fc12c4925d31/display/?preview=True#r1c2) | Terpene | 734,101 | 755,015 | [Carotenoid](https://mibig.secondarymetabolites.org/go/BGC0000636/1) | Terpene | 14% |
| [Region 3](https://usegalaxy.org/datasets/f9cad7b01a472135efd7fc12c4925d31/display/?preview=True#r1c3) | RRE-containing | 2,732,762 | 2,753,601 |  | | |
|  |  |  |  |  |  |  |
| *S. halotolerans* | | | | | | |
| **Region** | **Type** | **From** | **To** | **Most similar known cluster** | | **Similarity** |
| [Region 1.1](https://usegalaxy.org/datasets/f9cad7b01a4721354a664be88f54f2cd/display/?preview=True#r1c1) | T3PKS | 1,188,146 | 1,229,321 |  | | |
| [Region 1.2](https://usegalaxy.org/datasets/f9cad7b01a4721354a664be88f54f2cd/display/?preview=True#r1c2) | RiPP-like | 1,575,685 | 1,596,355 |  | | |
| [Region 3.1](https://usegalaxy.org/datasets/f9cad7b01a4721354a664be88f54f2cd/display/?preview=True#r3c1) | RiPP-like | 44,915 | 57,068 |  | | |
| [Region 5.1](https://usegalaxy.org/datasets/f9cad7b01a4721354a664be88f54f2cd/display/?preview=True#r5c1) | RaS-RiPP | 1 | 21,279 | [Thermophilin 1277](https://mibig.secondarymetabolites.org/go/BGC0000561/1) | RiPP:Lanthipeptide | 35% |
| [Region 7.1](https://usegalaxy.org/datasets/f9cad7b01a4721354a664be88f54f2cd/display/?preview=True#r7c1) | [T3PKS](https://docs.antismash.secondarymetabolites.org/glossary/#t3pks) | 77,534 | 118,712 |  | | |

Supplementary Table 3: Secondary metabolite biosynthesis gene clusters in genome data of consortium species using GECCO. Predicted biosynthetic products as determine by GECCO are given in respect to the species. NRP= non ribosomal peptide, RiPP= Ribosomally synthesized and post-translationally modified peptides.

|  | NRP | Polyketide | Terpene | RiPP | Unknown |
| --- | --- | --- | --- | --- | --- |
| *B.subtilis* | 4 | 1 |  |  | 1 |
| *S.capitis* | 1 |  |  | 1 |  |
| *A.radioresistens* |  |  |  |  |  |
| *M.luteus* |  |  |  |  |  |
| *P.antarctica* | 4 |  |  |  | 1 |
| *P.cyclohexanicum* | 1 |  | 1 |  |  |
| *S.halotolerans* |  |  |  | 2 |  |

## 1.2 Supplementary Figures

Supplementary Figure 1: Determination of CFU/mL of (A) *B. subtilis* and (B) *S. capitis* before co-cultivation in combination with each different species of the bacterial consortium and within the bacterial consortium.

Supplementary Figure 2: pH value of co-cultivation cpproaches of (A) *B: subtilis* and (B) *S. capitis*. pH was determined before (plain) and after (dashed) incubation of co-cultivation using pH-Fix 4.5-10.0 colour-fixed pH test strips (Macherey-Nagel, Germany). (A) Cultivation of *B. subtilis* as single species is given as “Single”. Pairwise co-cultivation combinations with *B. subtilis* are given as “& species name”. (B) Cultivation of *S. capitis* as single species is given as “Single” and pairwise combinations with *S. capitis* are given as “& species name”.

Supplementary Figure 3: Biofilm formation of single species and the bacterial consortium. The semi-quantitative crystal violet assay was performed to determine biofilm formation. The species were incubated statically at 37 °C for 24 h. Biofilm formation is proportional to absorption at 570 nm. TSYB was used as negative control. The experiment was repeated independently three times with three biological replicates each. Statistical significance was tested between biofilm formation of the respective species of the bacterial consortium and the bacterial consortium itself, and is indicated by asterisks (*=p≤0.05, **=p≤0.005).
